# Potential Anti-SARS-CoV-2 Prodrugs Activated by Phosphorylation and Their Role in the Aged Population

**DOI:** 10.3390/molecules28052332

**Published:** 2023-03-02

**Authors:** Vivek P. Chavda, Divya Teli, Pankti C. Balar, Dixa Vaghela, Hetvi K. Solanki, Akta Vaishnav, Lalitkumar Vora

**Affiliations:** 1 Department of Pharmaceutics and Pharmaceutical Technology, L. M. College of Pharmacy, Ahmedabad 380008, India; 2 Department of Pharmaceutical Chemistry, L. M. College of Pharmacy, Ahmedabad 380009, India; 3 Pharmacy Section, L. M. College of Pharmacy, Ahmedabad 380008, India; 4 School of Pharmacy, Queen’s University Belfast, 97 Lisburn Road, Belfast BT9 7BL, UK

**Keywords:** prodrug, aged population, remdesivir, molnupiravir, favipiravir, 2-Deoxy-D-glucose (2-DG)

## Abstract

The COVID-19 pandemic has flared across every part of the globe and affected populations from different age groups differently. People aged from 40 to 80 years or older are at an increased risk of morbidity and mortality due to COVID-19. Therefore, there is an urgent requirement to develop therapeutics to decrease the risk of the disease in the aged population. Over the last few years, several prodrugs have demonstrated significant anti-SARS-CoV-2 effects in in vitro assays, animal models, and medical practice. Prodrugs are used to enhance drug delivery by improving pharmacokinetic parameters, decreasing toxicity, and attaining site specificity. This article discusses recently explored prodrugs such as remdesivir, molnupiravir, favipiravir, and 2-deoxy-D-glucose (2-DG) and their implications in the aged population, as well as investigating recent clinical trials.

## 1. Introduction

Every corner of the world has been affected by the current pandemic, coronavirus disease 2019 (COVID-19) [1,2,3,4]. According to the WHO, approximately 66 million people worldwide have died from COVID-19 [5]. To combat COVID-19, scientists around the world have worked tirelessly to develop small-molecule drugs [3,4,5,6], traditional medicines [7,8,9,10,11,12,13], and vaccines [10,14,15,16]. Vaccination has been the primary method of COVID-19 management to date. Over 12 billion doses of vaccines have been administered worldwide [5]. Due to the rapid changes in the SARS-CoV-2 viral genome, there is a challenge to the efficacy of vaccines [17,18,19]. Although effective in attaining herd immunity among most of the population, they seem ineffective against new variants such as omicron, delta, deltacron, and many more [20,21,22,23]. This necessitates the development of a safe and potentially brand-new drug to combat the continuing epidemic [24,25]. At the cellular level, antiviral prodrugs seem to be more effective [26]. A prodrug is a molecule that is converted into active metabolites inside the body to attain maximum therapeutic value. The prodrug carries its astonishing activity in the masked form, which prolongs its duration of action and increases the pharmacokinetic properties [27]. According to our review of the literature, four prodrugs, remdesivir, molnupiravir, favipiravir, and 2-deoxy-D-glucose, are particularly well known for their roles in COVID-19 [28,29,30,31].

The most widely used medication during the COVID-19 pandemic has been remdesivir (RDV), an ester prodrug of G-5734 [32]. Currently, it is the only drug in the small molecule antiviral category which is approved by the US FDA to manage nonhospitalized high-risk COVID-19 patients [33]. It contains a single phosphate moiety that efficiently carries it through the biological membrane and is converted to the triphosphate form to perform its activity [34]. RDV acts by inhibiting the RNA-dependent RNA polymerase (RdRp) enzyme of SARS-CoV-2. It is a broad-spectrum antiviral molecule that has a good effect, with EC_50_ values of 0.77, 0.069, 0.012, and 0.090 against SARS-CoV-2, SARS-CoV, EBOV, and MERS-CoV, respectively [35]. A study with a modified prodrug of RDV on human plasma suggests that the modification improved its half-life to 77% after 4 h from 47% after 2 h [36]. Another drug that targets RdRp is molnupiravir, which is a prodrug of β-D-*N*4-hydroxycytidine [37]. It is under phase II/III trials [38]. A hamster model study concluded that molnupiravir, when administered with favipiravir, shows a synergistic effect [39]. In 2021, molnupiravir was provisionally stated as a first-line drug for the treatment of moderate COVID-19 patients in the UK [40]. Phospho-ribosylation of favipiravir, to its active form with triphosphate, inhibits RdRp [41]. A study suggested that the administration of the drug increases the G → A and C → T or C → U mutation, which reduces the viral titer [42]. Unlike the above three molecules, 2-deoxy-D-glucose (2-DG) works through different mechanisms. This prodrug is ideally used in anticancer and anti-viral actions [43]. Due to its structure being similar to glucose, cells absorb it, and it inhibits hexokinase, which is necessary for glycolysis, the production of energy. This reduces energy production, preventing the virus from nourishing itself [44]. Apart from this molecule, some molecules specifically target Mpro, such as PF-07304814 and GC-376 [45,46,47]. Other broad-spectrum prodrugs target RdRp, such as tenofovir [48], AT-527 [49], cyanorona-20 [50], and many more. All of these molecules have proven their effectiveness against COVID-19 in one way or another. The present article focuses on the synthesis and clinical trials of major prodrugs which are used in the treatment of COVID-19 in aged populations. It also covers the consequences observed during the trials.

## 2. Chemistry and Synthesis of Anti-SARS-CoV-2 Prodrugs

Prodrugs can be chemically classified into carrier-linked prodrugs and bioprecursor prodrugs [51]. A carrier-linked prodrug consists of an active drug linked to an enzymatically removable carrier group. It can further be subclassified as a bipartite prodrug, where the carrier molecule is directly attached to an active drug; a tripartite prodrug, where the carrier molecule is attached to an active drug via a spacer or linker; or a mutual prodrug (codrug), which consists of two active drugs attached to each other, usually synergistic, where one will play a carrier role for the other and vice versa [52]. Finally, a bioprecursor prodrug is a compound that can be metabolized enzymatically to the active drug by molecular modification. Bioprecursor prodrugs can be converted into active forms by different mechanisms, such as biological oxidation mediated by CYP450 enzyme systems, proton activation, hydrolysis, elimination, reduction, nucleotide activation, phosphorylation, sulfation, and decarboxylation [53]. Here, we have discussed the synthesis of promising anti-SARS-CoV-2 bioprecursor prodrugs that can be activated by nucleotide activation (remdesivir, molnupiravir, and favipiravir) and by phosphorylation (2-deoxy-D-glucose).

### 2.1. Synthesis of Remdesivir

**Scheme 1:**
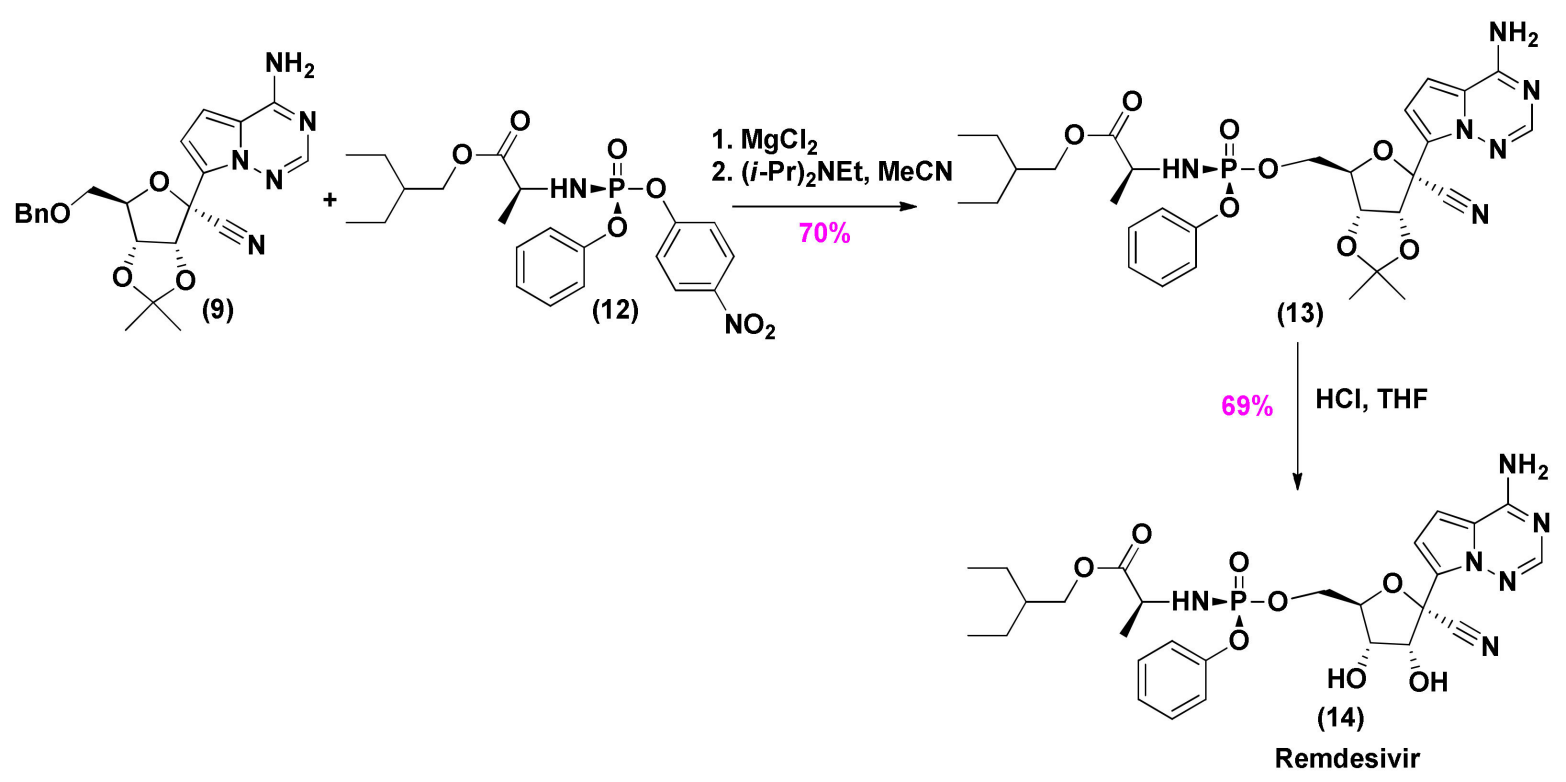
Final synthetic steps of Remdesivir, developed by Gilead.

**Scheme 2:**
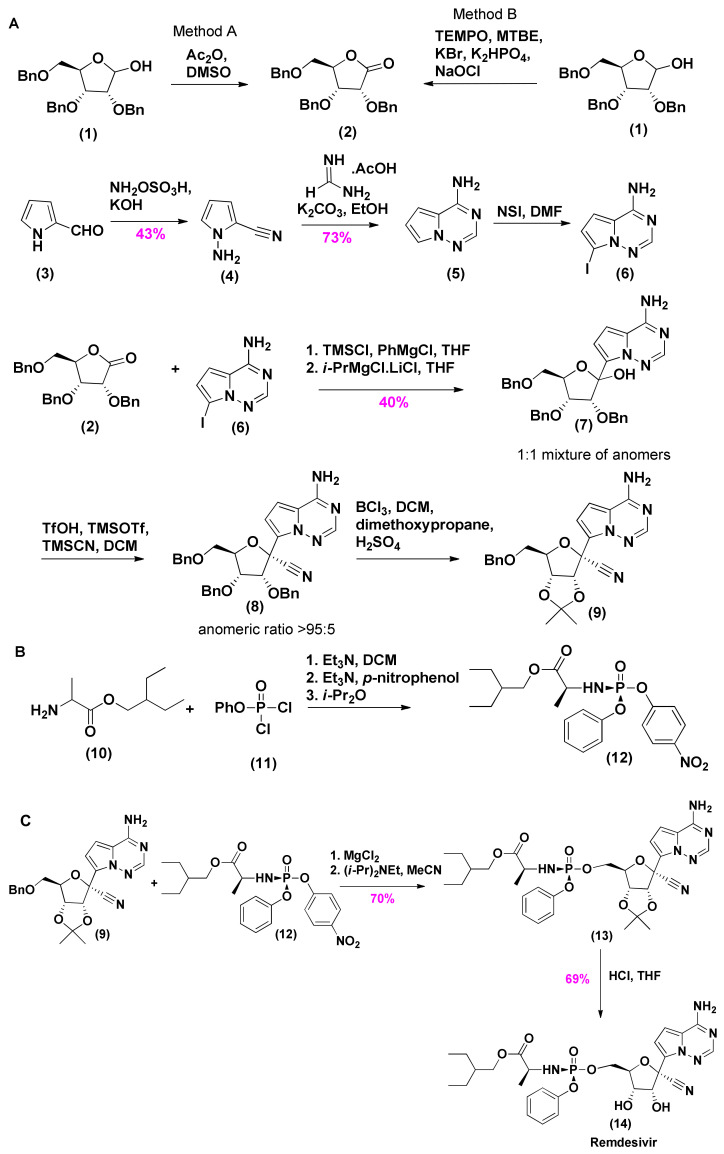
(**A**) Synthesis of the adenosine nucleoside analog (**9**) developed by Gilead; (**B*)*** Synthesis of *p*-nitrophenolate 2-ethyl butyl-L-alaninate (**12**); (**C**) Final synthetic steps of Remdesivir, developed by Gilead.

The complete synthetic route for Remdesivir was developed by Gilead biopharmaceuticals and involved the coupling of an adenosine nucleoside analog (**9**) with *p*-nitrophenolate 2-ethylbutyl-L-alaninate (**12**) (Scheme 1) [54]. The coupling was carried out in the presence of MgCl_2_ and *N*,*N*’-diisopropylethylenediamine base, followed by the deprotection of isopropylidene with HCl to afford Remdesivir. Tribenzylated ribose sugar (**1**) was first converted into ribolactone (**2**). Next, N-amination of pyrrole-2-carboxaldehyde (**3**) was carried out with hydroxylamine-*O*-sulfonic acid, followed by coupling with amidine and iodination to obtain 7-iodopyrrolotriazin-4-amine (**6**). Intermediates (**2**) and (**6**) were then treated with isopropyl magnesium chloride lithium chloride complex to generate a 1:1 anomeric mixture of intermediate (**7**), which was then subjected to cyanation using trimethylsilyl cyanide followed by acetonide protection to obtain adenosine nucleoside analog (**9**) (Scheme 2A). Finally, the *p*-nitrophenolate 2-ethylbutyl-L-alaninate precursor (**12**) was synthesized from 2-ethylbutyl-L-alanine (**10**) and phosphorodichloridate (**11**) using the sequence of reactions shown in Scheme 2B.

### 2.2. Synthesis of Molnupiravir

**Scheme 3:**
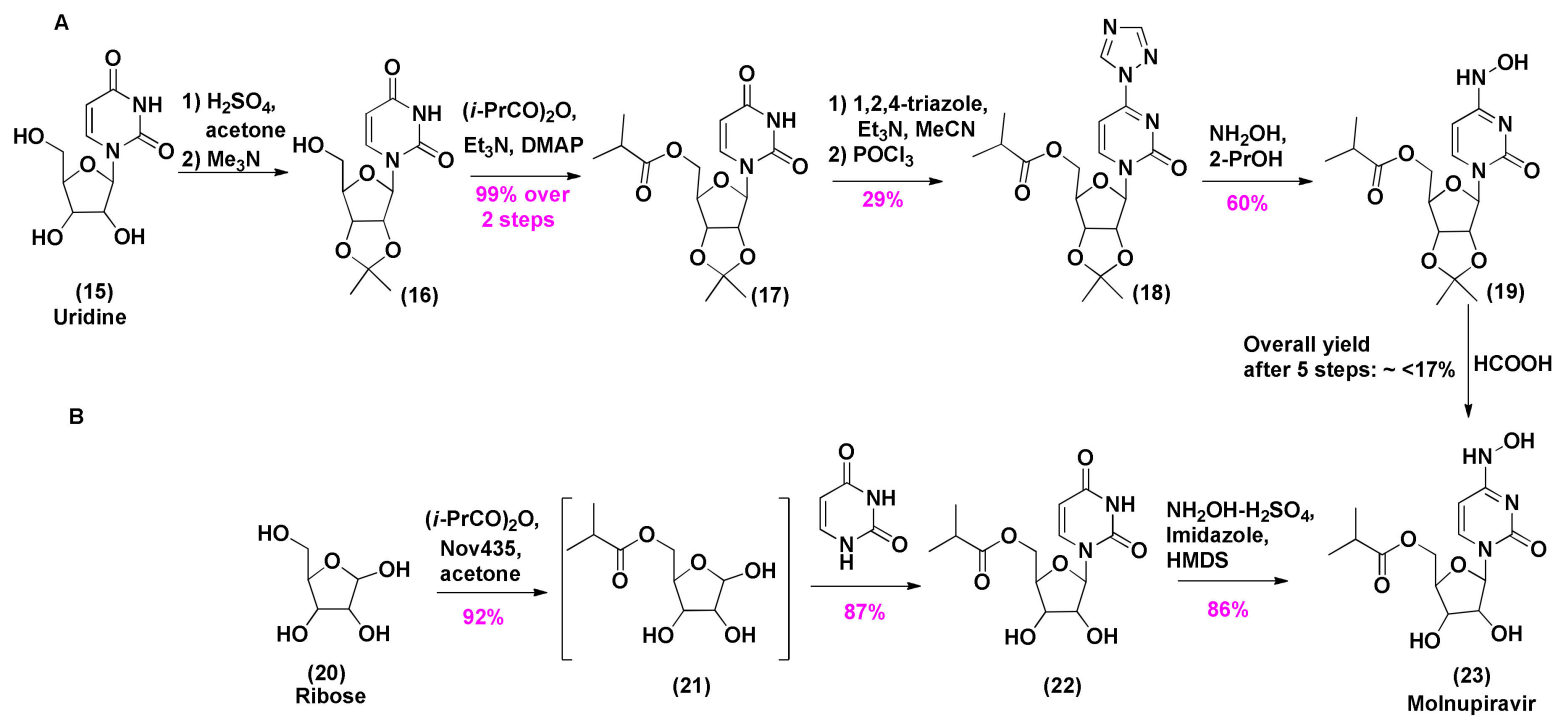
Synthesis of Molnupiravir. (**A**) The original synthetic route reported by Emory University used uridine as the starting material. (**B**) Potential synthetic route reported by Merck using ribose as the starting material.

Molnupiravir is a ribonucleoside prodrug composed of two structural fragments—*N*-hydroxycytosine and ribose sugar. Several synthetic routes to molnupiravir have recently been published using uridine, cytidine, or ribose as key starting materials [55]. Emory University was the first one to report a five-step synthetic method using uridine as a starting material, which has the drawback of very low yield and the use of the costly reagent 1,2,4-triazole (Scheme 3). This method starts with the synthesis of acetonide **16** by protecting the vicinal diol, followed by its reaction with isobutyric acid anhydride in the presence of 4-dimethylaminopyridine (DMAP) and triethylamine to obtain the corresponding isobutyryl ester **17**. The subsequent steps are chlorination, coupling with 1,2,4-triazole (**18**), the formation of hydroxylamine analog (**19**), and finally, the deprotection of acetonide using formic acid to afford molnupiravir. Unfortunately, the yield after the five steps was very poor [56].

To overcome the problems associated with the above scheme, a facile, high-yield, and low-cost commercial potential manufacturing route was developed by Merck and Codexis [57]. It consists of a three-step process containing ribose as a key starting material (Scheme 3A). Ribose was first esterified enzymatically. The next step required the usage of four enzymes—phosphorylation by MTR kinase, nucleobase formation by uridine phosphorylase, regeneration of acetyl phosphate and ATP by acetate kinase, and then the addition of pyruvate oxidase to generate the penultimate intermediate (**22**). In the last step, the intermediate (**22**) acidic carbonyl group was converted into hydroxylamine to afford molnupiravir.

### 2.3. Synthesis of Favipiravir

**Scheme 4:**
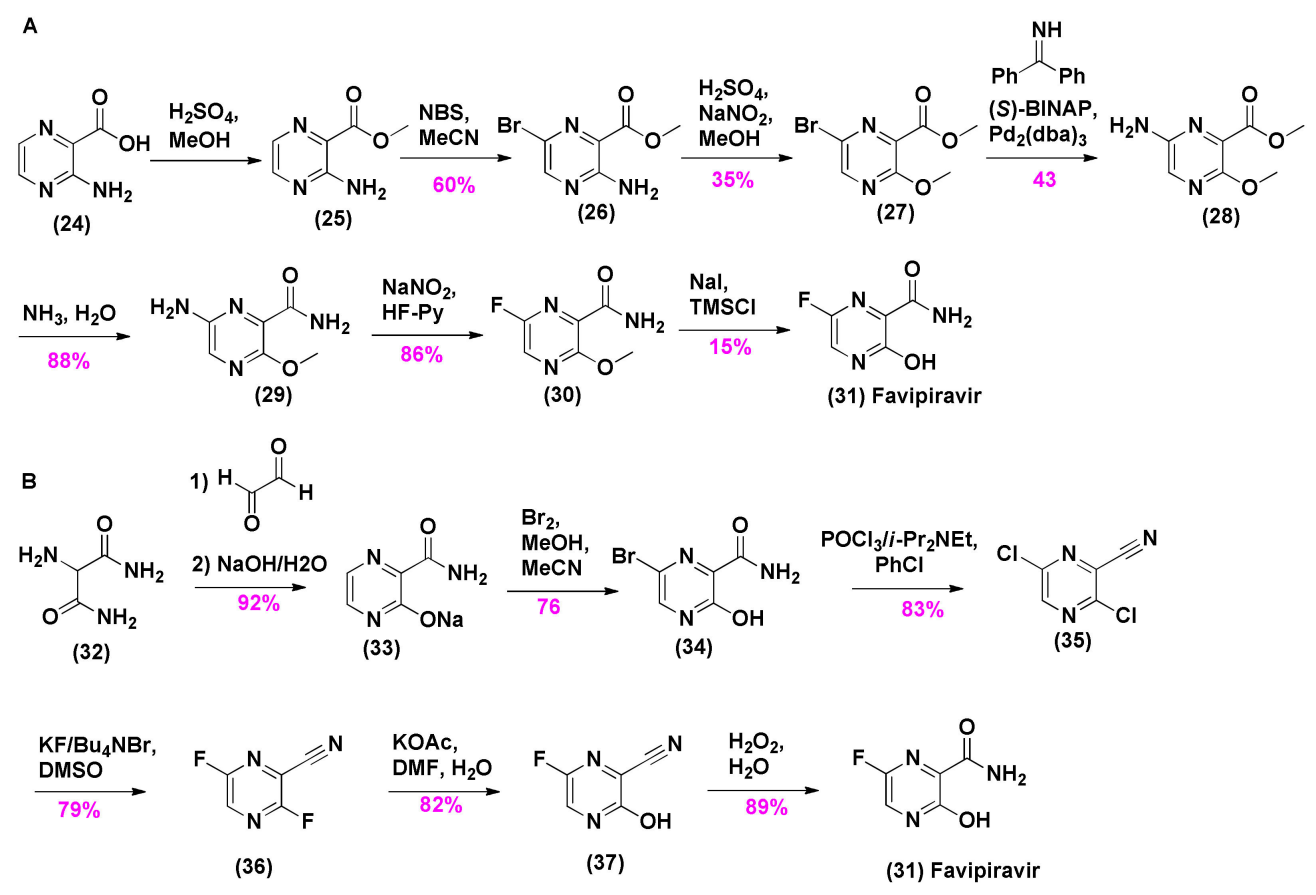
Synthesis of favipiravir. (**A**) Synthetic route developed by Toyama Company. (**B**) Potential synthetic route developed by Nippon Soda and Toyama Company.

The synthesis of favipiravir was first developed in 2000 by Furuta et al. from a Japanese company named Toyama Chemical Co., Ltd. [58]. Their method began with the synthesis of the methyl ester of 3-aminopyrazine-2-carboxylic acid (**24**), followed by bromination using *N*-bromosuccinimide, diazotization, alcoholysis, and Buchwald–Hartwig carbon–carbon bond coupling to synthesize the intermediate (**28**). Furthermore, aminolysis of the methyl ester yielded aminopyrazine carboxamide (**29**). Finally, the diazotization of the resultant intermediate (**29**), followed by treatment with Olah’s reagent (HF-pyridine) and demethylation of the methoxy group, afforded favipiravir (**31**). However, this method has the drawbacks of using a toxic reagent (Olah’s reagent), multistep synthesis, purification, and an overall very poor yield (Scheme 4A).

Toyama then collaborated with Nippon Soda to develop an improved and facile route that overcomes the problems associated with the earlier route (Scheme 4B) [59]. This six-step synthesis began with the reaction of 2-aminopropanediamide (**32**) with glyoxal in basic media to form the sodium salt of the intermediate (**33**). This was followed by bromination and subsequent chlorination, then reaction with *N*,*N*′-diisopropylethylamine in chlorobenzene at a reflux temperature to obtain 3,6-dichloro pyrazine-2-carbonitrile3,6-dichloro pyrazine-2-carbonitrile (**35**). This was then treated with a fluorinating agent to produce 3,6-difluoropyrazine-2-carbonitrile (**36**) and further reacted with potassium acetate and hydrogen peroxide to obtain favipiravir.

### 2.4. Synthesis of 2-Deoxy-D-Glucose

**Scheme 5:**
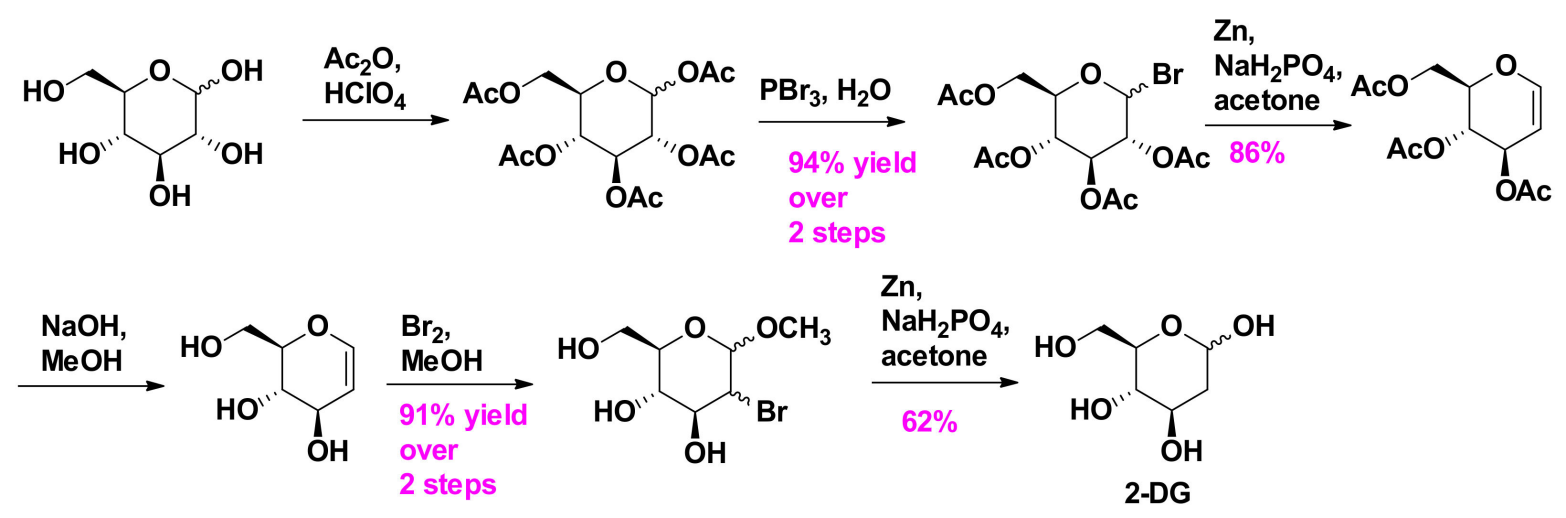
Synthesis of 2-Deoxy-D-Glucose.

Besides the recent use of 2-DG in COVID-19, it is effective against various other diseases, including cancer, dermatitis, polycystic kidney disease, and neurological disorders. Considering its promising therapeutic relevance, chemists have developed various strategies to synthesize 2-DG. The various routes of its synthesis have been previously reviewed and compiled by Marzabadi et al. [60] and Wijayasinghe et al. [61]. The methods that use inexpensive and readily available D-glucose as a starting material may be the most promising of all of the reported synthetic methods. W. Xu et al. reported a synthetic protocol which bears a 62% yield at the last step (Scheme 5) [62]. In this method, D-glucose is first acetylated to generate penta-*O*-acetyl-D-glucose, then treated with phosphorus tribromide (PBr_3_) to afford 1-bromo-2,3,4,6-tetra-*O*-acetyl-D-glucose. It is then reduced to triacetyl-*O*-D-glucal using Zn and monosodium phosphate (NaH_2_PO_4_). These steps are followed by subsequent hydrolysis, bromination, and reduction to obtain 2-DG with high yield and purity. Earlier, Vaidyanathaswamy et al. also reported the synthesis of 2-DG using D-glucose, with a 60% yield in the last step [63].

## 3. Clinical Trials of Anti-SARS-CoV-2 Prodrugs for Aged Populations

### 3.1. Remdesivir

**Figure 1:**
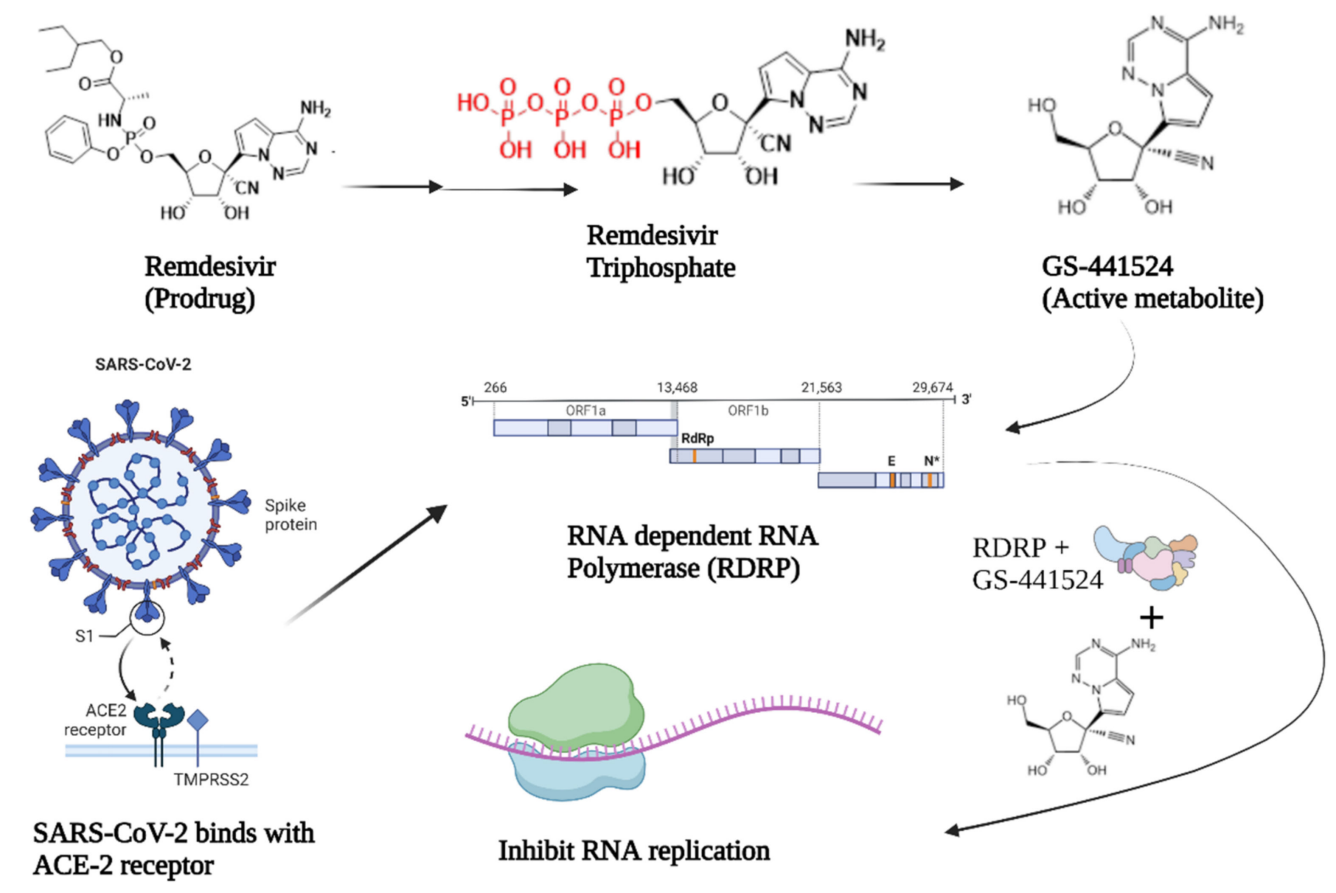
Mechanism of action of Remdesivir. It is converted into its nucleotide form and then into GS-441524, which inhibits RdRp of SARS-CoV-2. Abbreviations: SARS-CoV-2, severe acute respiratory syndrome coronavirus 2; ACE-2, angiotensin-converting enzyme 2.

The FDA authorized Remdesivir (Veklury) as the initial antiviral therapy for COVID-19 on 22 October 2020 [64]. A nucleoside analog under research named Remdesivir (GS-5734; Gilead Sciences Inc., Foster, CA, USA) functions as a competitive inhibitor of viral RdRp [65]. It is a prodrug of the adenosine nucleotide analog, which undergoes phosphorylation to form the nucleoside triphosphate analog after being converted to nucleoside monophosphate (Figure 1). With a precision mass of 602.23 Da and the molecular formula C_27_H_35_N_6_O_8_P, RdRp, a key enzyme for viral replication, interfaces with native ATP, and thus, is beneficial for the utilization of the nucleoside triphosphate derivative. Remdesivir is transformed within the body into GS-441524, an active molecule with the molecular formula C_12_H_13_N_5_O_4_ (291.10 Da) [66]. Mechanism of Remdesivir has been shown in Figure 1. 

Remdesivir should bind to SARS-CoV-2 RdRp with great affinity, according to molecular docking research, supporting its molecular mode of action [67]. Remdesivir has demonstrated low cytotoxicity in cultured cells and broad-spectrum antiviral efficacy by inhibiting the replication of several RNA viruses, including filoviruses, paramyxoviruses, pneumonia viruses, arenaviruses, and coronaviruses [68]. Several clinical trials are being conducted to fully evaluate the effectiveness of remdesivir in nations such as the USA, Norway, Canada, France, and China. Remdesivir is used in two dosages, with the first dose being 200 mg and the subsequent doses being 100 mg for the duration of the program. On 29 April 2020, the results of the first multicenter, randomized, double-blind, and placebo-controlled clinical trial were released [69]. The study’s main endpoint was the average time that a patient took to perceive a significant improvement. It involved 237 patients in China (158 received remdesivir and 79 received a placebo). The outcomes demonstrated that the time it took for a patient to see an improvement in symptoms was not substantially shortened by using remdesivir. Remdesivir may have provided few clinical benefits due to COVID-19 patients’ high mortality and slow virus clearance rates [70]. Additionally, there are not enough data on remdesivir, particularly in drug-gene and drug-disease intercommunication. Both in vitro and in vivo investigations have demonstrated its antiviral activity against SARS-CoV-2 [71,72]. Remdesivir has been prescribed as an emergency medication in several nations for COVID-19 patients, and some of these individuals exhibited better clinical results. However, extensive clinical trials must still be carried out to demonstrate the effectiveness of remdesivir in treating SARS-CoV-2 patients. Remdesivir effectively combatted novel varieties, including B.1.17 and B.1.351 [32,73,74]. In Table 1, some clinical trials which were performed in aged populations are listed.

The in vitro neutralization of omicron and its subvariants is still possible using remdesivir. Remdesivir can be completely infected by the omicron and delta versions of COVID-19. As a triphosphate metabolite, it directly inhibits the SARS-CoV-2 RdRp enzyme [74]. Andrea et al. studied the effect of remdesivir in people with SARS-CoV-2. It was administered to patients aged 40 years and older, with 60 patients in the control group and 18 people in the case group. The analysis showed that treatment with remdesivir and heparin led to lower mortality [75]. The SARS-CoV-2 variants have not been discovered to have any known ns12 mutations. Remdesivir is administered intravenously as an injection (100 mg) to COVID-19 adult and pediatric patients who must weigh at least 40 kg. However, significant contraindications have emerged, including anaphylaxis, hypersensitivity, shivering, bradycardia, dermatitis, nausea, etc. Remdesivir causes premature rupture, preterm birth, and fetal mortality; thus, pregnant women should not use it [76]. The most vulnerable group in society is elderly patients; however, the efficacy and safety of remdesivir for elderly patients have been checked in clinical trials. The efficacy of remdesivir in a real-world cluster of elderly patients hospitalized in Spain with COVID-19 was studied prior to the start of the vaccination programme. Per the study, patients under the age of 80 who received remdesivir had a 15.7% lower 30-day all-cause mortality rate and a 60% lower adjusted mortality risk than untreated patients. Even though they were most likely among those who should have received it, remdesivir was only given to a few patients with moderate to severe dependence and dementia [77]. Older patients had a lower chance of exhibiting raised severity scores than younger patients [78]. The risk of death for SARS-CoV2-infected individuals above the age of 80 is significantly higher than the risk for patients under the age of 80, according to recent epidemiological figures. Fever, cough, dyspnea, fatigue, and myalgia are the most common symptoms in elderly individuals, and only a small percentage of them develop acute respiratory distress syndrome (ARDS) [79].

### 3.2. Molnupiravir

**Figure 2:**
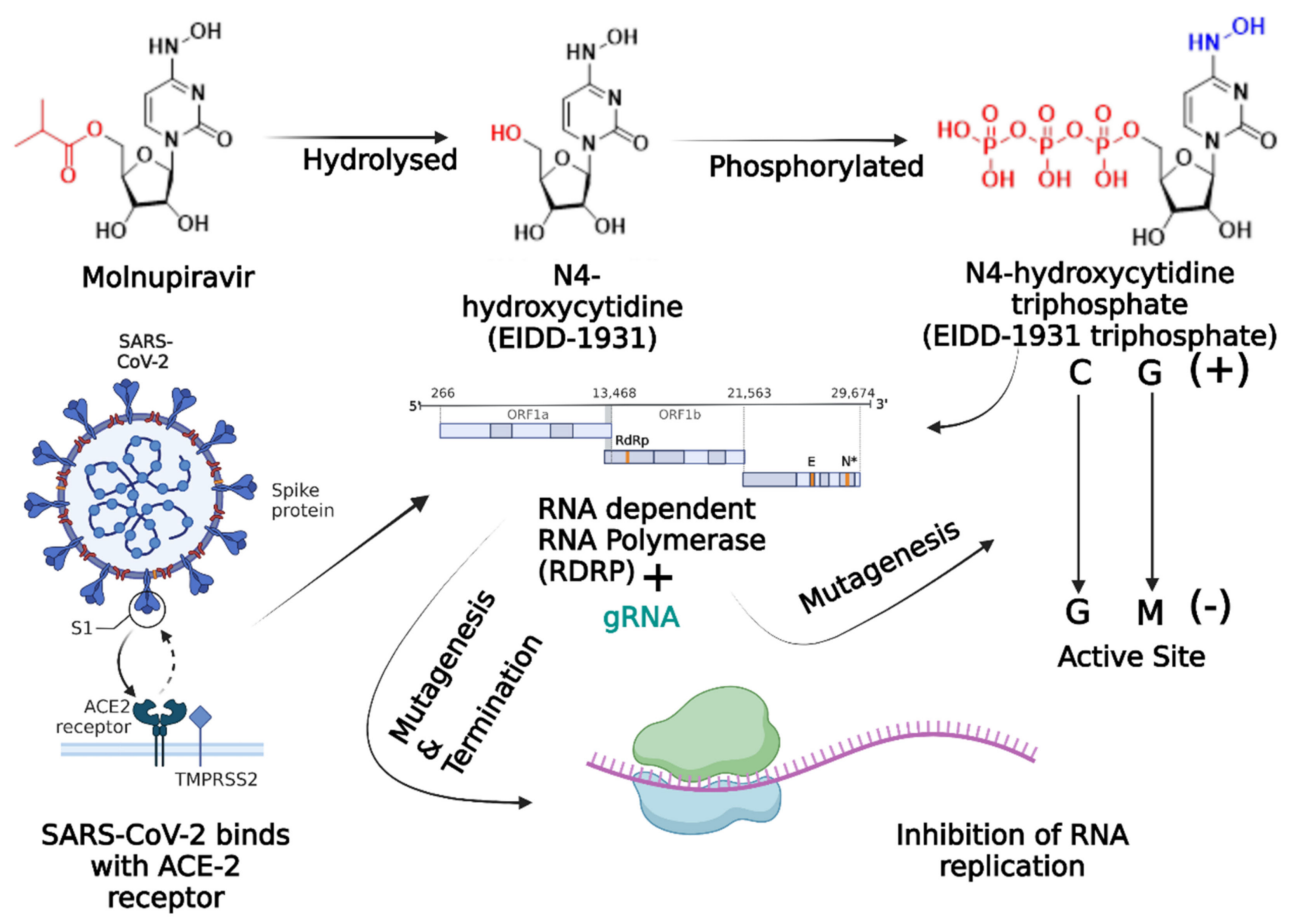
Mechanism of action of Molnupiravir. It is hydrolyzed to form NHC and then phosphorylated to form the nucleotide analog NHC-triphophate, which inhibits RdRp of SARS-CoV-2. Abbreviations: SARS-CoV-2, severe acute respiratory syndrome coronavirus 2; ACE-2, angiotensin-converting enzyme 2; C, cytidine triphosphate; G, guanosine triphosphate; M, N4-hydroxycytidine triphosphate.

The drug Molnupiravir (EIDD-2801) is the first orally available ribonucleoside which contains an isopropyl ester prodrug of *N*4-hydroxycytidine (NHC/EIDD-1931) with broad-spectrum antiviral activity against SARS-CoV-2 and other RNA viruses, as well as a high barrier to resistance development [80]. Similarly, to remdesivir, it also inhibits virus replication by inhibiting RdRp. It was first synthesized by the Emory Institute for Drug Development and was later taken up by Merck for further development [81]. Molnupiravir, a medicine which was co-developed by Merck and Ridgeback Biotherapeutics, was revealed on 1 October 2021. It can significantly diminish the risk of hospitalization or mortality in patients with mild or moderate COVID-19, which has sparked great interest in the drug among the scientific community [82]. Several studies have elucidated the mechanisms of Molnupiravir, emphasizing its critical role in the suppression of SARS-CoV-2 infection [83,84]. First, Molnupiravir is hydrolyzed to *N*4-hydroxycytidine and then phosphorylated to *N*4-hydroxycytidine triphosphate (MTP) with the help of host kinases (Figure 2). MTP can cause mutations in viral synthesis and inactivate virus offspring by participating in viral replication and increasing the G to A and C to U substitutions [40]. A normal complementary base pairing would pair G with C (G:C), and A would pair with U or T (A: U/A: T), while MTP could pair either with A or G. After being integrated into the template strand, C: G or U: A can moderately impede the extension of MTP: G or MTP: A products, although this inhibition can be decreased when the concentration of MTP is sufficiently high [83]. As a result of the strong antiviral action of Molnupiravir, mutations from G to A (G: NHC: A) and C to U (C: G: NHC: A: U) have also been observed in subsequent replication [84]. Detailed mechanism of action of Molnupiravir has been give in Figure 2. In addition, Angelica Jayk Bernal and coworkers analyzed the effect of Molnupiravir as an oral treatment in non-hospitalized patients. They concluded that early treatment with Molnupiravir will reduce the risk of death and hospitalization in unvaccinated patients with COVID-19 [80].

Molnupiravir has been tested in preclinical studies as an orally bioavailable nucleoside analog prodrug of NHC. Being a prodrug, it demonstrates enhanced stability. Furthermore, it exhibits higher oral bioavailability in ferrets, nonhuman primates, and mice than the prototype metabolite NHC. [85,86]. In addition, according to preclinical studies, Molnupiravir has shown a low risk of genotoxicity [83,84,87]. Table 2 describes the ongoing clinical trials on Molnupiravir.

Molnupiravir provides broad antiviral action against various genetically unrelated coronaviruses, including the recently identified SARS-CoV-2 variants of concern (alpha, beta, gamma, delta, and omicron) [86,88]. Recent research has revealed that Molnupiravir can suppress the omicron variant both in vitro and in vivo [89,90]. Nevertheless, the data on its effectiveness and safety in the omicron-infected population are still insufficient. Andrea et al. studied the safety and efficacy of molnupiravir in 192 patients with a mean age from 70.4 to 15.4 years among 144 patients over 60 years. The early onset of antiviral treatment avoids disease progression and reduces comorbidities such as cardiovascular disease, chronic lung disease, obesity, and diabetes. In addition, molnupiravir has no interaction with other chronic treatments [91]. One study assessed the efficacy of molnupiravir in hospitalized COVID-19 patients. Among 11,822 patients, molnupiravir was administered to 203 patients, whereas 387 did not receive any antiviral therapy and thus required dexamethasone and baricitinib therapy. Treatment with molnupiravir reduced mortality, and it was evident in patients over 80 years of age that were treated in the first five days of the disease that molnupiravir therapy did not affect the frequency of need for ventilation. Nevertheless, patients treated with molnupiravir required oxygen supplementation less frequently [92]. Molnupiravir was effective when provided orally to adults aged 80 and up, potentially enhancing the results. Furthermore, this medicine efficiently stopped human transmission of the SARS-CoV-2 virus in hospital wards. There were no deaths within 30 days, demonstrating its effectiveness. Only 55% of the target patients experienced mild adverse events within seven days of internal administration. Only nausea was noted; no other serious side effects were observed, indicating that the medicine can be safely given to elderly individuals. Early Molnupiravir treatment reduced the likelihood of death in older people at risk for COVID-19. Because the sample size was so small, it was insufficient as reliable evidence [93].

### 3.3. Favipiravir

**Figure 3:**
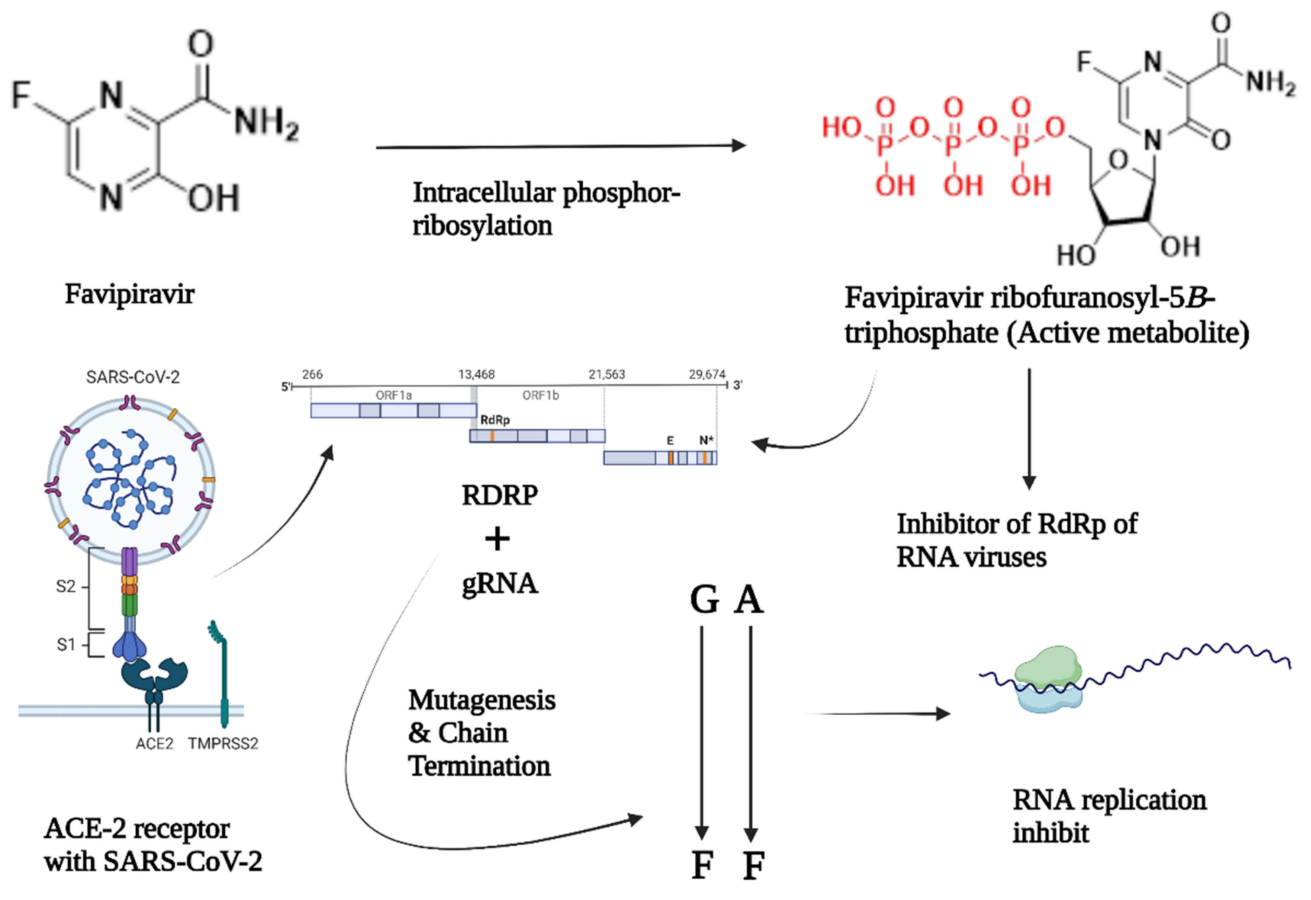
Mechanism of action of favipiravir. It is phosphoribosylated to afford its active nucleotide analog, which inhibits RdRp of SARS-CoV-2. Abbreviations: SARS-CoV-2, severe acute respiratory syndrome coronavirus 2; ACE-2, angiotensin-converting enzyme 2; RdRP, RND-dependent RNA polymerase; gRNA, guide RNA.

Favipiravir is a prodrug with antiviral properties and is used to treat COVID-19. It has a low molecular weight of 157.1 g/mol [94]. Favipiravir is a purine base analog; after oral administration, favipiravir is converted into its active metabolite, favipiravir ribofuranosyl-5*B*-triphosphate (Favipiravir-RTP), by intracellular phosphor-ribosylation [95]. It acts as a selective inhibitor of RdRp of RNA viruses. Favipiravir has a broad spectrum of antiviral properties that enable various RdRps in several types of RNA viruses. The favipiravir RTP adds pressure on coronavirus nucleotides, which already have low cytosine in their SARS-CoV-2 genome. With an increased frequency of mutation, favipiravir RTP has a positive effect on the virus cytopathic effect, which induces a reduction in viral RNA. It has a long binding affinity to RdRp; hence, it targets SARS-CoV-2 and ultimately prevents viral transcription and replication [96]. Mechanism of action of Favipiravir has been shown in Figure 3.

The effect of favipiravir on SARS-CoV-2-infected people was studied by performing a placebo-controlled phase 2 trial. Before starting this trial, a non-randomized study of favipiravir in patients aged 18 and above were found the drug to exhibit more than a 90% viral clearance [97]. The clinical trial data found that favipiravir was beneficial over a placebo regarding secondary progression in infected patients. Upon oral administration, it inhibited viral replication, and its pharmacokinetic properties also act on the virologic activity [98].

Another randomized, open-label phase 3 clinical study was undertaken in patients aged 18–75 years to determine the safety and efficacy of the drug. For oral administration of favipiravir, RT–PCR negativity was 28.7% earlier in moderate COVID-19 patients. The antiviral effect was 40% faster than that of a placebo, and it was found to be safe and well-tolerated in open-label clinical trials [99]. Yuan et al. performed another study to evaluate favipiravir’s clinical efficacy and safety in patients with severe fever with thrombocytopenia syndrome (SFTS). A total of 23,350 patients aged < 70 years, between 60 and 70 years, and >70 years were involved in the study. Favipiravir significantly reduced case fatality rates from 20% to 9%; however, a significant effect was observed among patients aged <70 years with a therapy duration of >5 days. Instant favipiravir therapy could help SFTS patients aged 60–70 years; however, these therapies cause hyperuricemia and thrombocytopenia in >70-year-old patients [100]. A clinical study was conducted in China to study favipiravir’s effectiveness against SARS-CoV-2 patients, especially in elderly individuals that were 65 years and older. Patients received 1600 mg twice a day on day 1, and 600 mg twice a day from day 2 to day 14, and the primary outcome was monitored by computed tomography to evaluate the efficiency of the drug [101]. Ivashcenko et al. conducted an open-label study with 60 patients that were above 60 years of age or older. Patients received 1800–1600 mg on the first day and 800–600 mg on the second day, which resulted in decreased viral load and normalized body temperature, and approximately 62.5% and 92.5% of patients had a negative PCR result on day 5 and day 10, respectively [102].

Patients received either a placebo or 1800 mg of favipiravir BID (two times a day) on day 1 and 800 mg of favipiravir BID from days 2 to 13. Although there were more patients over 65 years of age in the placebo group, there were higher- or medium-risk patients in the favipiravir group. A 2020 observational study by Fujita Health University found that symptoms deteriorated more frequently in patients 60 and older than in younger patients and that senior patients saw improvements in fewer cases than younger individuals. The numbers of patients in trials testing favipiravir as a COVID-19 medication were modest, and a tiny percentage of people that were 65 and older took part in the trials [103,104].

A recent in vitro study by Wang et al. reported that favipiravir and oseltamivir combination therapy might facilitate clinical recovery in patients and reduce SARS-CoV-2 infection [105]. The viral clearance rate for AVIFAVIR versus the standard of care in hospitalized SARS-CoV-2 patients was nearly 62.5% when investigated in phase 2 and phase 2/3 trials. Two different doses, 1600/600 mg and 1800/800 mg, were given and showed similar virologic responses. On day 10, viral clearance was achieved in 92.5% of patients who received AVIFAVIR and 80% of patients who received SOC [106]. Further analysis and safety data were obtained from phase 2 and 3 clinical studies of favipiravir. It could be reliable and endurable in a short span of use; however, more evidence is required for longer-term treatments [107]. Table 3 describes the ongoing trials on favipiravir.

### 3.4. 2-Deoxy-D-Glucose

**Figure 4:**
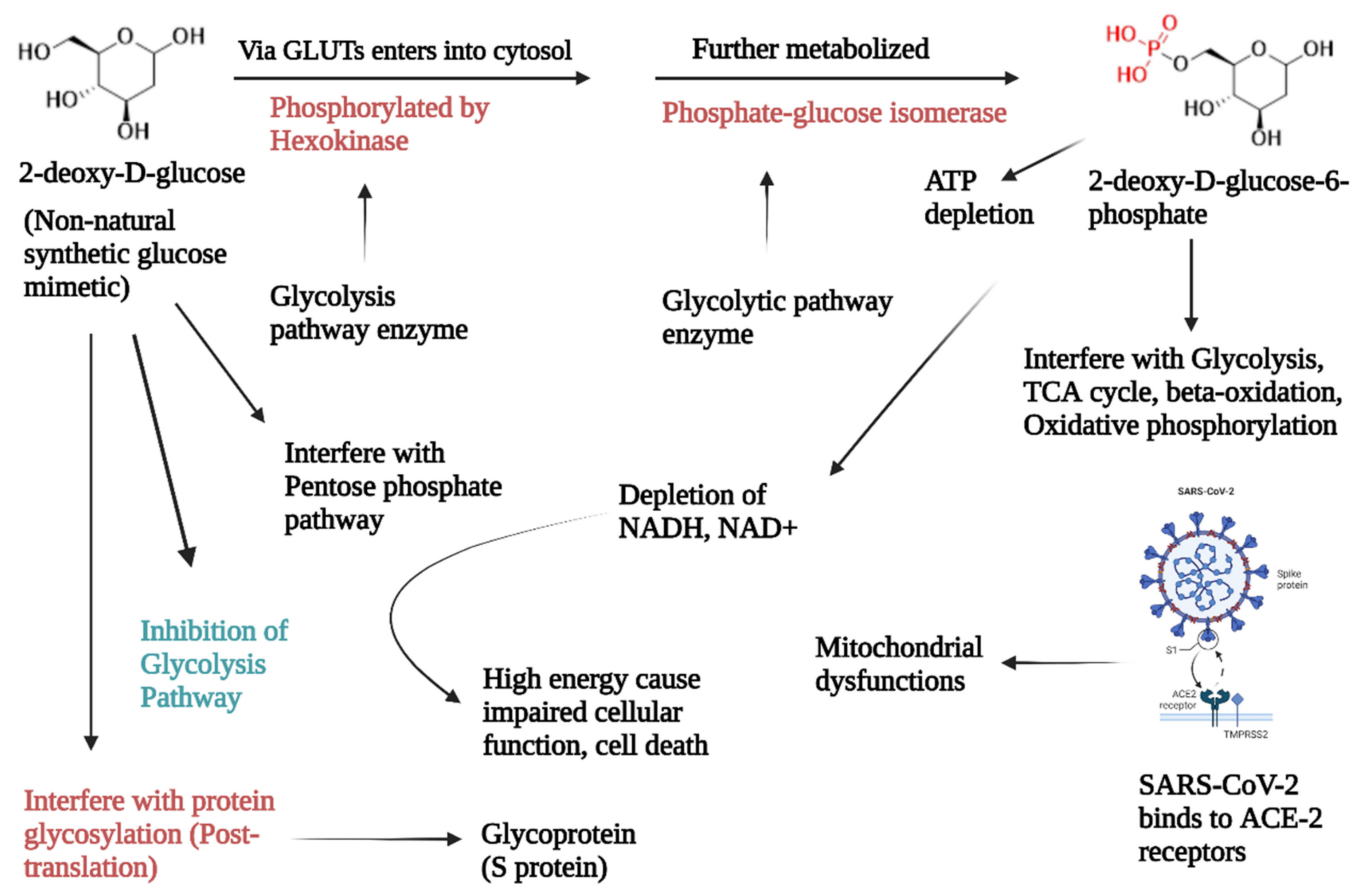
Mechanism of action of 2-Deoxy-D-glucose. It is converted into its active form, 2-deoxy-D-glucose, which depletes cellular ATP. Abbreviations: SARS-CoV-2, severe acute respiratory syndrome coronavirus 2; ACE-2, angiotensin-converting enzyme 2; TCA, tricarboxylic acid cycle; ATP, adenosine triphosphate; S protein, spike protein.

2-DG is a glucose analog which is effective as an antiviral agent and was approved by the Indian Council of Medical Research (ICMR) in May 2021 for the treatment of SARS-CoV-2 infection. It is essentially a glucose molecule in which the 2-hydroxyl group is replaced by hydrogen [108]. The glucose analog enters the cytosol and is phosphorylated to 2-DG-6P (2-deoxy-D-glucose-6-phosphate) by the enzyme hexokinase, which is further metabolized by phosphate-glucose isomerase, leading to the accumulation of 2-DG-6P in cells and the depletion of cellular ATP [31]. Specifically, in infected lung cells, it decreases glycolysis and energy production and inhibits viral replication. Thus, the cell survival rate decreases and the cell death pathway is activated [109]. Further, 2-DG is more stable than its active metabolite, 2-DG-6P, and has a longer shelf life. The drug 2-DG-6P is a potent inhibitor of glycolysis, which is a central metabolic pathway in cells. The direct administration of 2-DG-6P can result in systemic toxicity. In Figure 4, Detailed information has been shown with their mechanism of action. However, by administering 2-DG as a prodrug, the cells and tissues regulate the conversion to the active metabolite, which can help to minimize toxicity. The Drug Controller General of India (DCGI) approved its emergency use as an adjuvant therapy in coronavirus patients on 17 May 2021. It reduces viral load and the need for oxygen supplement therapy [110].

Preliminary in vitro studies have shown that 2-DG can potentially reduce the viral load in host cells [111]. Vero and Vero E6 cell lines were used as in vitro model systems to study the effect of fluorescent 2-DG against SARS-CoV-2 infection. It was discovered that 2-DG treatment of Vero cells compromised SARS-CoV-2’s infection and multiplication efficiency, reducing the ability to infect newer cells and showing overall effective inhibition of virus growth [112]. In addition, it was discovered that 2-DG possessed a binding energy of −140.05 kcal/mol with 3CLpro. Furthermore, bioanalytical and toxicity studies suggest that 2-DG produces sufficient oral bioavailability without side effects or toxicity [113].

Moreover, 2-DG also has multiple pharmacological effects and controls SARS-CoV-2 infection by improving the host immune response. According to a fractional-order optimal control problem (FOCP) study, 2-DG generates better results in preventing and controlling SARS-CoV-2 infection [114]. In addition, Verma et al. concluded that 2-DG could enhance the efficacy of low-dose radiation therapy (LDRT) in COVID-19 pneumonia patients by preventing lung damage. Furthermore, low doses could decrease the cytokine storm by inducing anti-inflammatory responses [115]. Table 4 describes the ongoing clinical trials on 2-DG.

The Institute of Nuclear Medicine and Allied Sciences (INMAS) and a DRDO scientist initiated phase 2 clinical trials in May 2020 [116]. The phase 2 study found substantial recovery progress in 2.5 median days in normalizing vital parameters between the 2-DG arm and progress to the SoC arm. A further phase 3 trial enrolled various Indian states and was recently approved in May 2021 by the DCGI for the emergency use of 3-DG as an adjoining therapy in patients with moderate to severe COVID-19 [117]. A randomized, open-label phase 2 clinical trial evaluated the efficacy, safety, and tolerability of 2-DG. A total of 110 patients between the ages of 18 and 65 with moderate to severe COVID-19 were involved in this study. A dose of 63 mg/kg/day, 90 mg/kg/day, or 126 mg/kg/day of 2-DG was given to the patients; among these doses, 90 mg/kg/day showed a beneficial effect in the treatment of severe to moderate COVID-19 [118].

## 4. Impact of Prodrugs on Vaccinated COVID-19 Patients

Various treatment options have been investigated to prevent the catastrophic progression of COVID-19 in communities. Vaccinated patients have also been monitored regarding the use of antiviral prodrugs to reduce the progression of disease symptoms. Recently, a matched-pair retrospective study was conducted to assess the role of remdesivir and vaccination in preventing severe clinical outcomes [119]. The nonhospitalized vaccinated patients who received a three-day course of remdesivir had a 75% lower risk of hospitalization and a 95% lower risk of respiratory failure. In addition, the study concluded that, in high-risk, nonhospitalized patients, vaccination combined with a three-day course of remdesivir significantly prevented severe clinical progression of COVID-19.

To evaluate the efficacy of another antiviral prodrug, molnupiravir, a phase 2 experimental study called AGILE CST-2 was conducted in vaccinated and unvaccinated COVID-19 patients [120]. The study included 180 participants with an average age of 43 years. Most of them had already received their first dose of the COVID-19 vaccine. The experiment included patients with alpha (37 of 180), delta (72 of 180), omicron (38 of 180), and EU1 (28 of 180) variants. Participants in the molnupiravir group received negative PCR results faster (8 days) than those in the placebo group (11 days). Thus, molnupiravir was found to have antiviral activity in vaccinated and unvaccinated individuals infected with a wide range of SARS-CoV-2 variants, although this evidence is inconclusive.

A longitudinal study covering the role of vaccine, favipiravir, and micronutrient supplementation was conducted to evaluate post-COVID symptoms [121]. The study included 923 people (18.2% were fully vaccinated). One of the most frequently reported post-COVID symptoms was fatigue. The combination of micronutrients such as vitamins C and D and favipiravir did not affect all post-COVID symptoms. When infected with SARS-CoV-2, a full COVID-19 vaccination prevents the disease and the development of residual symptoms. As a result, it is critical to reconsider the prescription of micronutrients or adjust the dose of favipiravir in the current guidelines.

## 5. Conclusions and Future Perspectives

The scientific community has made significant progress in generating potential antiviral medications; however, effective anti-SARS-CoV-2 therapies are still not accessible. COVID-19 can be treated effectively with broad-spectrum anti-SARS-CoV-2 prodrugs. Aging generates various biochemical changes in the immune system which have been associated with age-related disorders and susceptibility to infectious infections. The likelihood of developing severe symptoms increases with age, with people aged 40 and older having the greatest risk of developing major symptoms. Several prodrugs have demonstrated significant anti-SARS-CoV-2 effects in vitro, in animal models, and in medical practice; their effectiveness in avoiding mortality is limited. Various factors should be considered for prodrug treatment, such as modes of drug administration, optimized combination treatment, prodrug-based lead optimization, and rationalized doses. Because of its rapid viral clearance, improved clinical recovery rate, and availability as an oral medicine with an evidenced safety profile, it is a promising treatment which can be repurposed to treat COVID-19. All prodrugs used for the treatment of aged people reduce the risk of death and hospitalization in unvaccinated and vaccinated patients with COVID-19. Therefore, all prodrugs are suggested for use in patients with the highest risk of hospital admission. Continuing clinical trials on prodrugs worldwide will provide further information on their clinical effectiveness, safety, and therapeutic role in the overall care of COVID-19.

All potential anti-SARS-CoV-2 prodrugs that are activated by phosphorylation play an important role in the treatment of COVID-19. In the majority of systematic reviews, including meta-analyses, remdesivir was not confirmed to be the best antiviral for SARS-CoV-2 infection; nevertheless, a reduction in hospitalization and adverse events in COVID-19 patients was demonstrated. Remdesivir did not significantly reduce mortality or the need for mechanical ventilation, especially compared to a placebo. Furthermore, additional pharmacological trials are being conducted to examine the molecule’s efficacy in treating COVID-19. In vitro studies of favipiravir have shown high efficacy against COVID-19 infection using concentrated and safe therapeutic doses. Favipiravir encourages quicker viral clearance, treatment outcomes, and enhanced radiological imaging in hospitalized patients. However, it may not be beneficial for those that are not hospitalized. Monitoring hyperuricemia while taking favipiravir will help to ensure that the medication is terminated as soon as possible. Molnupiravir is only required for a short period (five days) and is easier to administer in an outpatient setting, resulting in higher compliance. The benefit of molnupiravir is that it may be manufactured on a larger scale and does not require cold transportation or administration in hospital settings, unlike other EUA-approved COVID-19 medicines. However, it remains unclear whether molnupiravir is a “miracle” drug. The drug 2-DG was approved for emergency use by the Indian drug authority. It accumulates in virus-infected cells, inhibiting viral reproduction by preventing viral synthesis and energy output. Establishing an online data repository for recording the efficacy and side effects of 2-DG would be tremendously useful in combining data on 2-DG administration in COVID-19. To confirm the early findings, a multicentric study with a larger population and from other geographical regions is needed.

## Figures and Tables

**Table 1:** Clinical trials on remdesivir have focused more on aged populations.

Other ID	Study Type	Study Starts	End of Study	Phase	Age Group	Remarks	NCT No.
CAP-China Remdesivir 2	Interventional	6 February 2020	10 April 2020	3	≥18 years,	237 participants Primary outcome: six death; five ICU, requiring extracorporeal membrane oxygenation (IMV), needed Noninvasive ventilation (NIV) therapy; three Hospitalization, requiring respirator oxygen (but not NIV/HFNC); two hospitalizations, no need for additional oxygen, one discharge with clinical meetup Secondary outcome: ventilation system for 28 days, continuous oxygenation, admission in hospital until recovery upper and lower respiratory tract samples are 2019-nCoV RT–PCR negativity	NCT04257656
LGH003	Interventional	1 June 2020	30 November 2020	Early phase 1	15 to 80 years	30 participants Primary outcome (10 days): measure the permeability of drug, concentration of drug into the body, volume of distribution Secondary outcome (15 days): the general prognosis of COVID-19 patients following medication therapy, duration spent in the hospital, whether in the ICU or in HDUs, until the date of discharge, time that the need for respiratory distress has expanded from the beginning	NCT04560231
BEX-06001	Interventional	4 September 2020	30 April 2021	2	≥18 years	60 participants Primary outcome (28 days): measure expanded of hospital stay days and clinical Secondary outcome (28 days): relieves and encounters diseases, does not require more treatment, does not require ventilator support (but not NIV/HFNC therapy), ICU/hospitalization, needing ECMO and/or IMV, death, endotracheal, oxygen supplementation, time to 2019 nCoV RT–PCR negativity in the nasopharyngeal swab, the incidence of significant serious adverse reactions, death rate ended up causing	NCT04596839
COVID 19 treatment	Interventional	16 June 2020	1 December 2020	2, 3	Older adults,	200 participants Primary outcome (6 months): number of patients with improvement or mortality	NCT04345419
REC-H-PhBSU-21001	Interventional	1 November 2020	1 April 2021	4	18 to 80 years	90 participants (recruiting)	NCT04738045
UW 20-535	Interventional	20 November 2020	30 September 2021	2	Above 18 years	Primary outcome (30 days): Clinical improvement Secondary outcome: hospitalization (30 days), nasopharyngeal swab viral load (7 days), evaluate mediators (7 days), adverse events (5 days), mortality (30 days)	NCT04647695
M.A.R.M.C.D./2020/1985	Interventional	15 August 2020	10 February 2021	3	16 to 80 years	205 participants Primary outcome (30 days): clinical Improvement Secondary outcome (30 days): duration of ICU stays, mortality rate, recovery time, the effectiveness of oxygen capacity, period taken in failure of clinical study	NCT04678739
GS-US-553-9020	Interventional	14 September 2020	22 March 2021	1, 2	Above 18 years	156 participants Primary outcome (7 days): average weight loss, saliva, nasopharyngeal and oropharyngeal weight has difference due to SARS-CoV-2 viral load Secondary outcome (5 to 30 days): Number of participants including all physiologically supervised consultations or death, proportion of people who have experienced therapies abnormalities, proportion of participants undergoing diagnosis adverse reactions, proportion of participants to treatment-emergent adverse reactions leading to study treatment cessation, and pharmacokinetic criteria	NCT04539262
FMASU P56a/2020	Interventional	27 July 2020	1 March 2021	3	18 to 80 years	77 participants Primary outcome (14 days): evaluation of viral clearance	NCT04853901
REM-ENY-01	Interventional	12 October 2020	30 November 2021	3	12 to 100 years	2000 participants Primary outcome (30 days): To evaluate the safety and acceptability of employing the tentatively authorized prescription for those who are desperate for food Secondary outcome (30 days): percentage of patients who experienced at least 1 treatment-related unexpected reaction, the percentage of patients who experienced a treatment-related illness	NCT04610541
CAP-China Remdesivir 1	Interventional	12 February 2020	27 April 2020	3	Above 18 years	Suspended	NCT04252664
DW_DWJ1248302	Interventional	2 February 2021	30 June 2021	3	19 years and older	1022 participants Primary outcome (29 days): Rate of mortality Secondary outcome (29 days): Measure days spent recovering, rankings of acceptable outcomes, hospitalization period, death ratio	NCT04713176
VC-02-01	Interventional	16 June 2020	1 December 2020	2	18 years and older	44 participants Primary outcome (28 days): count of participants who were hospitalized but did not have pulmonary collapse or other serious effects Secondary outcome: Frequency of sickness, the percentage of fatalities, whether continuous breathing was necessary and for how long, the use of antihypertensive medications, oxygen therapy, the amount of demand for oxygen, and the end of primary infection are all factors	NCT04410354
GS-US-540-5773-2020-000841-15 ISRCTN1587426	Interventional	6 March 2020	30 June 2020	3	Above 12 years	4891 participants Primary outcome (14 days): proportion of volunteers classified by clinical state on a basis of 7-point rating variable Secondary outcome (10 days): respondents encountered therapies adverse outcomes as a frequency	NCT04292899
NEUROSIVIR	Interventional	15 September 2020	31 March 2021	1	21 to 50 years	45 participants Primary outcome: percentage of patients who experienced any treatment-related side effect, as well as categorized laboratory results Secondary outcome: extended of area under curve with infinite space, elevated value within the duration of required time, distributed volume, clearance	NCT04480333
GS-US-540-9012 2020-003510-12	Interventional	18 September 2020	6 May 2021	3	Above 12 years	584 participants Primary outcome (28 days): percentage of participants Secondary outcome: time-weighted average change, time to alleviation, required oxygen supplementation	NCT04501952
GS-US-540-5774 2020-000842-32 ISRCTN85762140	Interventional	15 March 2020	26 June 2020	3	Above 12 years	1113 participants Primary outcome (11 days): proportion of individuals characterized by the clinical state on a 7-point ordinary scale Secondary outcome (40 days): volunteers who encountered therapies and a fraction of abnormalities	NCT04292730
GS-US-540-5912 2020-005416-22 DOH-27-012022-4779	Interventional	31 March 2021	24 May 2022	3	Above 12 years	249 participants Primary outcome (29 days): measure intrusive ventilation system or a mixture of all-cause mortality Secondary outcome: renal replacement therapy, time to recover, the mortality rate	NCT04745351
SOL21	Interventional	24 July 2021	31 December 2023	4	Above 18 years	202 participants Primary outcome (1 year): recovery, fatigue, exertional dyspnea, long-COVID symptoms Secondary outcome (2 years): recovery, fatigue, exertional dyspnea, long-COVID symptoms	NCT04978259
RECH-PhBSU-21011	Interventional	1 October 2020	5 April 2021	4	18 to 88 years	150 participants Primary outcome (5-7 days): percentage of clinical cure	NCT04779047

Abbreviations: ECMO—extracorporeal membrane oxygenation, IMV—intermittent mandatory ventilation, NIV—noninvasive ventilation, HFNC—high flow nasal cannula, nCoV RT–PCR—novel coronavirus reverse transcription polymerase chain reaction, ICU—intensive care unit, HDU—high dependency unit.

**Table 2:** Clinical trials on molnupiravir have focused more on aged populations.

Other ID	Study Type	Study Start Date	Study Completion Date	Phase	Age Group	Remarks	NCT No.
EIDD-2801-2003	Interventional	19 June 2020	21 February 2021	Phase 2	18 years and older	204 participants Primary estimated outcome estimates the time required for SARS-CoV-2 clearance in nasopharyngeal Swabs Secondary outcome-Determine Molnupiravir’s safety and tolerability	NCT04405570
EZ-SS-029	Interventional	12 August 2022	July 2027	Phase 3	50 years and older	4000 participants Primary outcome—evaluate the safety, effectiveness, and adverse effect of Molnupiravir in adults with mild COVID-19 Secondary outcome—evaluate tolerability and the association between the efficacy of Molnupiravir and the period between the onset of symptoms and the start of therapy	NCT05459532
MOL-112021	Interventional	1 December 2021	11 March 2022	Phase 3	18 Years to 80 years	240 participants Primary outcome—the prevalence of patients with COVID-19 severity has increased from baseline. Secondary outcome—score the severity of symptoms using the COVID-19 Major Symptom Rating Scale.	NCT05595824
4482-001 MK-4482-001 PHRR201210-003189 jRCT2031200404 2020-003367-26	Interventional	19 October 2020	11 August 2021	Phase 2 Phase 3	18 years and older	304 participants Primary outcome—evaluate the time required for the sustained recovery and adverse effect Secondary outcome—measure the rate of mortality	NCT04575584
4482-002 MK-4482-002 PHRR201209-003186 jRCT2031210148 2020-003368-24	Interventional	19 October 2020	5 May 2022	Phase 2/3	18 years and older	1734 participants Primary outcome—% of participants hospitalized and/or dying and having a negative impact Secondary outcome—time to long-term remission or improvement of each specified COVID-19 sign/symptom and progression	NCT04575597
4482-013 MK-4482-013 jRCT2031210281 PHRR211007-003980 2021-000904-39	Interventional	11 August 2021	17 November 2022	Phase 3	18 years and older	1376 participants Primary outcome—% of individuals with undetectable SARS-CoV-2, ≥1 adverse event, and withdrawal from study treatment owing to AE Secondary outcome— % of subjects with detectable and undetectable SARS-CoV-2	NCT04939428
EIDD-2801-2004	Interventional	16 June 2020	21 February 2022	Phase 2	18 years and older	71 participants Primary outcome—the number of participants who achieve virologic clearance and have severe Adverse Events (SAEs) after taking Molnupiravir orally. Secondary outcome	NCT04405739
NL78705.018.21	Observational	14 December 2021	14 June 2024	-	18 years and older	1000 participants Primary outcome—treatment with monoclonal antibodies and antivirals changes serologic responses Secondary outcome—the therapeutic impact of monoclonal antibodies and antiviral medicines, as well as the incidence of treatment-emergent adverse effects	NCT05195060
UoL001542	Interventional	3 July 2020	30 April 2024	Phase 1 Phase 2	18 years and older	600 participants Primary outcome—determine the safety, ability, and tolerability Secondary outcome—assess clinical progress	NCT04746183
MOL-05-02-2021	Interventional	1 April 2022	31 December 2023	Not Applicable	18 years to 45 years	50 participants Primary outcome—evaluate the Cmax, tmax, AUC0-t (area under curve), AUC0-inf, AUCextr, t1/2, kel, MRT, ratio of Cmax, ratio of AUC0-t, and ratio of AUC0-inf Secondary outcome—evaluate safety and tolerability	NCT05412173
NDPHRECOVERY 2020-001113-21 ISRCTN50189673	Interventional	19 March 2020	November 2032	Phase 2 Phase 3	0 years and older	50,000 participants Primary outcome—evaluate causes of mortality Secondary outcome—assess the composite death endpoint or the requirement for mechanical ventilation	NCT04381936
COVIC-19	Interventional	1 April 2022	10 September 2024	Phase 3	Child, adult, older adult	680 participants Primary outcome—proportion of subjects hospitalized with COVID-19 symptoms that worsened or died Secondary outcome—the percentage of patients who require supplementary oxygen, noninvasive ventilation, intubation, or mechanical ventilation.	NCT05271929
ASC10-102	Interventional	28 November 2022	9 October 2023	Phase 1	18 years and older	32 participants Primary outcome—the number of individuals who experienced a Treatment-Emergent Adverse Event (TEAEs) and measured PK parameters for Molnupiravir Secondary outcome—time (days) to complete resolution of all COVID-19 signs/symptoms	NCT05596045
PR005	Observational	1 December 2021	30 September 2027	-	18 years to 50 years	2000 participants Primary outcome—examine obstetric and neonatal outcomes, infant weight and height, and developmental milestones in infants Secondary outcome	NCT05013632
VIR21001	Interventional	30 September 2021	August 2024	Phase 2	18 years to 50 years	1500 participants Primary outcome—positive controls and viral clearance rates for newly available and repurposed medicines Secondary outcome—viral kinetics in early COVID-19 illness, the number of antiviral treatment arms, and viral clearance rates	NCT05041907
EIDD-2801-1001 2020-001407-17	Interventional	10 April 2020	11 August 2020	Phase 1	18 years to 60 years	130 participants Primary outcome—number of treatment participants, adverse events and treatment severity unexpected adverse events Secondary outcome	NCT04392219

**Table 3:** Clinical trials on favipiravir have focused more on aged populations.

Other ID	Study Type	Study Start Date	Study Complication Date	Phase	Age Group	Remark	NCT Number
Favipiravir-A	Interventional	16 February 2021	13 July 2021	Phase 3	50 years and older	500 participants Primary outcome: need of oxygen supplement Secondary outcome: there were changes in ICU stay and mortality rate	NCT04818320
CONTROL-COVID-Favipiravir-1	Interventional	16 October 2020	November 2021	Phase 2	65 years and older	760 participants Primary outcome: after treatment, there were no new cases of COVID-19 until 25 days. Secondary outcome: improvement in common symptoms after 40 days of administration.	NCT04448119
FMASU P14/2020	Interventional	18 April 2020	20 June 2020	Phase 3	18 to 80 years	100 participants Primary outcome: viral clearance observed within 14 days. Normal body temperature and two successive negative tests were analyzed. Secondary outcome: improvement of radiological abnormalities in patients.	NCT04349241
RC 20/220/R	Interventional	23 July 2020	4 August 2021	phase 2 and 3	18 years and older	231participenats. Primary outcome: negative RT–PCR test within 15 days. Secondary outcome: Normal symptoms were normalized within 72 h of administration. The severity of disease complications was decreased within 28 days.	NCT04464408
FAV-052021	Interventional	11 August 2021	30 December 2021	phase 3	18 years to 80 years	217 participants	NCT05185284
FAV052020	Interventional	21 May 2020	20 August 2020	phase 3	18 years to 80 years	200 participants	NCT04542694
FAVI-COV-US201	Interventional	17 April 2020	30 October 2020	Phase 2	18 years to 80 years	50 participants Primary outcome: at 29 days decrease in viral clearance. Secondary outcome: respiration rate, oxygen saturation, pulse rate, and systolic blood pressure.	NCT04358549
CVD-04-CD-001	Interventional	22 August 2020	27 January 2021	Phase 3	21 to 70 years	353 patients involved. Primary outcome: After 1–28 days of treatment, resolution of hypoxia in the patient and the score of RT–PCR is improved. Secondary outcome: hospital discharge time interval was shorter after treatment.	NCT04529499
COVID-19-FAV-HQ	Interventional	16 November 2020	16 February 2021	Phase 3	18 to 59 years	1120 participants Primary outcome: worsening of clinical findings Secondary outcome: complete resolution in symptoms and signs	NCT04981379
Favipiravir covid	Interventional	20 April 2020	28 September 2020	Phase 2 and phase 3	18 to 80 years	96 participants (divided into 2 groups: favipiravir and chloroquine, each having 48 participants) Clinical outcomes: patients treated with favipiravir had shorter hospital stays and did not require mechanical ventilation compared to those treated with Chloroquine.	NCT04351295

**Table 4:** Clinical trials on 2-DG have focused more on aged populations.

Other ID	Study Type	Study Start Date	Study Complication Date	Phase	Age Group	Remarks	NCT Number
COMPOSIT Study	Interventional	4 January 2021	4 January 2022	Not applicable	40 years and older	50 participants This study was conducted on 220 COVID patients; 42% of patients improved symptomatically and became free from depending on oxygen supplementation.	NCT05009563

## Data Availability

Not applicable.

## References

[1] Piret J., Boivin G. (2021). Pandemics Throughout History. Front. Microbiol..

[2] Chavda V.P., Vora L.K., Pandya A.K., Patravale V.B. (2021). Intranasal Vaccines for SARS-CoV-2: From Challenges to Potential in COVID-19 Management. Drug Discov. Today.

[3] Chavda V.P., Soni S., Vora L.K., Soni S., Khadela A., Ajabiya J. (2022). MRNA-Based Vaccines and Therapeutics for COVID-19 and Future Pandemics. Vaccines.

[4] Chavda V.P., Yao Q., Vora L.K., Apostolopoulos V., Patel C.A., Bezbaruah R., Patel A.B., Chen Z.-S. (2022). Fast-Track Development of Vaccines for SARS-CoV-2: The Shots That Saved the World. Front. Immunol..

[5] WHO Coronavirus (COVID-19) Dashboard. https://covid19.who.int.

[6] De Savi C., Hughes D.L., Kvaerno L. (2020). Quest for a COVID-19 Cure by Repurposing Small-Molecule Drugs: Mechanism of Action, Clinical Development, Synthesis at Scale, and Outlook for Supply. Org. Process Res. Dev..

[7] Lei S., Chen X., Wu J., Duan X., Men K. (2022). Small Molecules in the Treatment of COVID-19. Signal Transduct. Target. Ther..

[8] Laws M., Surani Y.M., Hasan M.M., Chen Y., Jin P., Al-Adhami T., Chowdhury M., Imran A., Psaltis I., Jamshidi S. (2021). Current Trends and Future Approaches in Small-Molecule Therapeutics for COVID-19. Curr. Med. Chem..

[9] Liu H., Iketani S., Zask A., Khanizeman N., Bednarova E., Forouhar F., Fowler B., Hong S.J., Mohri H., Nair M.S. (2022). Development of Optimized Drug-like Small Molecule Inhibitors of the SARS-CoV-2 3CL Protease for Treatment of COVID-19. Nat. Commun..

[10] Alam S., Sarker M.M.R., Afrin S., Richi F.T., Zhao C., Zhou J.-R., Mohamed I.N. (2021). Traditional Herbal Medicines, Bioactive Metabolites, and Plant Products Against COVID-19: Update on Clinical Trials and Mechanism of Actions. Front. Pharmacol..

[11] Teli D.M., Shah M.B., Chhabria M.T. (2021). In Silico Screening of Natural Compounds as Potential Inhibitors of SARS-CoV-2 Main Protease and Spike RBD: Targets for COVID-19. Front. Mol. Biosci..

[12] Demeke C.A., Woldeyohanins A.E., Kifle Z.D. (2021). Herbal Medicine Use for the Management of COVID-19: A Review Article. Metab. Open.

[13] Umeta Chali B., Melaku T., Berhanu N., Mengistu B., Milkessa G., Mamo G., Alemu S., Mulugeta T. (2021). Traditional Medicine Practice in the Context of COVID-19 Pandemic: Community Claim in Jimma Zone, Oromia, Ethiopia. Infect. Drug Resist..

[14] COVID-19 Vaccine Resource Center. https://www.nejm.org/covid-vaccine.

[15] Moghadas S.M., Vilches T.N., Zhang K., Wells C.R., Shoukat A., Singer B.H., Meyers L.A., Neuzil K.M., Langley J.M., Fitzpatrick M.C. (2021). The Impact of Vaccination on Coronavirus Disease 2019 (COVID-19) Outbreaks in the United States. Clin. Infect. Dis..

[16] Forni G., Mantovani A., on behalf of the COVID-19 Commission of Accademia Nazionale dei Lincei, Rome (2021). COVID-19 Vaccines: Where We Stand and Challenges Ahead. Cell Death Differ..

[17] Mistry P., Barmania F., Mellet J., Peta K., Strydom A., Viljoen I.M., James W., Gordon S., Pepper M.S. (2022). SARS-CoV-2 Variants, Vaccines, and Host Immunity. Front. Immunol..

[18] Krause P.R., Fleming T.R., Longini I.M., Peto R., Briand S., Heymann D.L., Beral V., Snape M.D., Rees H., Ropero A.-M. (2021). SARS-CoV-2 Variants and Vaccines. N. Engl. J. Med..

[19] Planas D., Veyer D., Baidaliuk A., Staropoli I., Guivel-Benhassine F., Rajah M.M., Planchais C., Porrot F., Robillard N., Puech J. (2021). Reduced Sensitivity of SARS-CoV-2 Variant Delta to Antibody Neutralization. Nature.

[20] The Effects of Virus Variants on COVID-19 Vaccines. https://www.who.int/news-room/feature-stories/detail/the-effects-of-virus-variants-on-covid-19-vaccines.

[21] Chavda V.P., Apostolopoulos V. (2022). Is Booster Dose Strategy Sufficient for Omicron Variant of SARS-CoV-2?. Vaccines.

[22] Chavda V.P., Apostolopoulos V. (2022). Global Impact of Delta plus Variant and Vaccination. Expert Rev. Vaccines.

[23] Chavda V.P., Bezbaruah R., Deka K., Nongrang L., Kalita T. (2022). The Delta and Omicron Variants of SARS-CoV-2: What We Know So Far. Vaccines.

[24] Chavda V.P., Mohapatra S.S., Ranjan S., Dasgupta N., Mishra R.K., Thomas S. (2019). Nanobased Nano Drug Delivery: A Comprehensive Review. Applications of Targeted Nano Drugs and Delivery Systems.

[25] Chavda V.P., Mohapatra S.S., Ranjan S., Dasgupta N., Mishra R.K., Thomas S. (2019). Nanotherapeutics and Nanobiotechnology. Applications of Targeted Nano Drugs and Delivery Systems.

[26] Wang Z., Yang L. (2022). Broad-spectrum Prodrugs with Anti-SARS-CoV-2 Activities: Strategies, Benefits, and Challenges. J. Med. Virol..

[27] Rautio J., Meanwell N.A., Di L., Hageman M.J. (2018). The Expanding Role of Prodrugs in Contemporary Drug Design and Development. Nat. Rev. Drug Discov..

[28] Yasri S., Wiwanitki V. (2022). Molnupiravir, Favipiravir and Other Antiviral Drugs with Proposed Potentials for Management of COVID-19: A Concern on Antioxidant Aspect. Int. J. Biochem. Mol. Biol..

[29] Hashemian S.M.R., Pourhanifeh M.H., Hamblin M.R., Shahrzad M.K., Mirzaei H. (2022). RdRp Inhibitors and COVID-19: Is Molnupiravir a Good Option?. Biomed. Pharmacother..

[30] Kulkarni P., Padmanabhan S. (2022). A Novel Property of Hexokinase Inhibition by Favipiravir and Proposed Advantages over Molnupiravir and 2 Deoxy d Glucose in Treating COVID-19. Biotechnol. Lett..

[31] Huang Z., Chavda V.P., Vora L.K., Gajjar N., Apostolopoulos V., Shah N., Chen Z.-S. (2022). 2-Deoxy-D-Glucose and Its Derivatives for the COVID-19 Treatment: An Update. Front. Pharmacol..

[32] Eastman R.T., Roth J.S., Brimacombe K.R., Simeonov A., Shen M., Patnaik S., Hall M.D. (2020). Remdesivir: A Review of Its Discovery and Development Leading to Emergency Use Authorization for Treatment of COVID-19. ACS Cent. Sci..

[33] Center for Drug Evaluation and Research (2021). FDA’s Approval of Veklury (Remdesivir) for the Treatment of COVID-19—The Science of Safety and Effectiveness.

[34] Yang L., Wang Z. (2021). Natural Products, Alone or in Combination with FDA-Approved Drugs, to Treat COVID-19 and Lung Cancer. Biomedicines.

[35] Wang Z., Yang L. (2020). GS-5734: A Potentially Approved Drug by FDA against SARS-Cov-2. New J. Chem..

[36] El-Sayed N.S., Jureka A.S., Edwards M.R., Lohan S., Williams C.G., Keiser P.T., Davey R.A., Totonchy J., Tiwari R.K., Basler C.F. (2021). Synthesis and Antiviral Activity of Fatty Acyl Conjugates of Remdesivir against Severe Acute Respiratory Syndrome Coronavirus 2 and Ebola Virus. Eur. J. Med. Chem..

[37] Li Y., Liu M., Yan Y., Wang Z., Dai Q., Yang X., Guo X., Li W., Chen X., Cao R. (2022). Molnupiravir and Its Active Form, EIDD-1931, Show Potent Antiviral Activity against Enterovirus Infections In Vitro and In Vivo. Viruses.

[38] Wang Z., Yang L. (2020). Turning the Tide: Natural Products and Natural-Product-Inspired Chemicals as Potential Counters to SARS-CoV-2 Infection. Front. Pharmacol..

[39] Abdelnabi R., Foo C.S., Kaptein S.J.F., Zhang X., Do T.N.D., Langendries L., Vangeel L., Breuer J., Pang J., Williams R. (2021). The Combined Treatment of Molnupiravir and Favipiravir Results in a Potentiation of Antiviral Efficacy in a SARS-CoV-2 Hamster Infection Model. EBioMedicine.

[40] Syed Y.Y. (2022). Molnupiravir: First Approval. Drugs.

[41] Agrawal U., Raju R., Udwadia Z.F. (2020). Favipiravir: A New and Emerging Antiviral Option in COVID-19. Med. J. Armed. India.

[42] Baranovich T., Wong S.-S., Armstrong J., Marjuki H., Webby R.J., Webster R.G., Govorkova E.A. (2013). T-705 (Favipiravir) Induces Lethal Mutagenesis in Influenza A H1N1 Viruses in Vitro. J. Virol..

[43] Kang H.T., Hwang E.S. (2006). 2-Deoxyglucose: An Anticancer and Antiviral Therapeutic, but Not Any More a Low Glucose Mimetic. Life Sci..

[44] Sahu K.K., Kumar R. (2021). Role of 2-Deoxy-D-Glucose (2-DG) in COVID-19 Disease: A Potential Game-Changer. J. Fam. Med. Prim. Care.

[45] Sharun K., Tiwari R., Dhama K. (2021). Protease Inhibitor GC376 for COVID-19: Lessons Learned from Feline Infectious Peritonitis. Ann. Med. Surg..

[46] Owen D.R., Allerton C.M.N., Anderson A.S., Aschenbrenner L., Avery M., Berritt S., Boras B., Cardin R.D., Carlo A., Coffman K.J. (2021). An Oral SARS-CoV-2 Mpro Inhibitor Clinical Candidate for the Treatment of COVID-19. Science.

[47] Narayanan A., Narwal M., Majowicz S.A., Varricchio C., Toner S.A., Ballatore C., Brancale A., Murakami K.S., Jose J. (2022). Identification of SARS-CoV-2 Inhibitors Targeting Mpro and PLpro Using in-Cell-Protease Assay. Commun. Biol..

[48] Lee M.J., Nori A., Fidler S., Kulasegaram R., Fox J., Smith C. (2022). RECEDE-C19 study Response to ‘The Use of Tenofovir in Patients with COVID-19’. HIV Med..

[49] Shannon A., Fattorini V., Sama B., Selisko B., Feracci M., Falcou C., Gauffre P., El Kazzi P., Delpal A., Decroly E. (2022). A Dual Mechanism of Action of AT-527 against SARS-CoV-2 Polymerase. Nat. Commun..

[50] Rabie A.M. (2021). Cyanorona-20: The First Potent Anti-SARS-CoV-2 Agent. Int. Immunopharmacol..

[51] Jornada D.H., dos Santos Fernandes G.F., Chiba D.E., de Melo T.R.F., dos Santos J.L., Chung M.C. (2015). The Prodrug Approach: A Successful Tool for Improving Drug Solubility. Molecules.

[52] Rautio J., Kumpulainen H., Heimbach T., Oliyai R., Oh D., Järvinen T., Savolainen J. (2008). Prodrugs: Design and Clinical Applications. Nat. Rev. Drug Discov..

[53] Testa B. (2009). Prodrugs: Bridging Pharmacodynamic/Pharmacokinetic Gaps. Curr. Opin. Chem. Biol..

[54] Siegel D., Hui H.C., Doerffler E., Clarke M.O., Chun K., Zhang L., Neville S., Carra E., Lew W., Ross B. (2017). Discovery and Synthesis of a Phosphoramidate Prodrug of a Pyrrolo[2,1-f][Triazin-4-Amino] Adenine C-Nucleoside (GS-5734) for the Treatment of Ebola and Emerging Viruses. J. Med. Chem..

[55] Hughes D.L. (2021). Quest for a Cure: Potential Small-Molecule Treatments for COVID-19, Part 2. Org. Process Res. Dev..

[56] Painter G.R., Guthrie D.B., Bluemling G.R., Natchus M.G. (2016). N4-Hydroxycytidine and Derivatives and Anti-Viral Uses Related Thereto 2016. WO Patent.

[57] Benkovics T., McIntosh J., Silverman S., Kong J., Maligres P., Itoh T., Yang H., Huffman M., Verma D., Pan W. (2020). Evolving to an Ideal Synthesis of Molnupiravir, an Investigational Treatment for COVID-19. ChemRxiv.

[58] Furuta Y., Egawa H. (2000). Nitrogenous Heterocyclic Carboxamide Derivatives or Salts Thereof and Antiviral Agents Containing Both. WO Patent.

[59] Hara T., Norimatsu N., Kurushima H., Kano T. (2014). Method for Producing Dichloropyrazine Derivative. U.S. Patent.

[60] Marzabadi C.H., Franck R.W. (2000). The Synthesis of 2-Deoxyglycosides: 1988–1999. Tetrahedron.

[61] Wijayasinghe Y.S., Bhansali M.P., Borkar M.R., Chaturbhuj G.U., Muntean B.S., Viola R.E., Bhansali P.R. (2022). A Comprehensive Biological and Synthetic Perspective on 2-Deoxy-d-Glucose (2-DG), A Sweet Molecule with Therapeutic and Diagnostic Potentials. J. Med. Chem..

[62] Xu W., Yang H., Liu Y., Hua Y., He B., Ning X., Qin Z., Liu H.-M., Liu F.-W. (2017). Facile Approaches to 2-Deoxy-d-Glucose and 2-Deoxy-α-d-Glucopyranonucleosides from d-Glucal. Synthesis.

[63] Indian Patents. 187908: “An Improved Process for Preparation of 2-Deoxy-D-glucose”. https://www.allindianpatents.com/patents/187908-an-improved-process-for-preparation-of-2-deoxy-d-glucose.

[64] Commissioner of the FDA Approves First Treatment for COVID-19. https://www.fda.gov/news-events/press-announcements/fda-approves-first-treatment-covid-19.

[65] Warren T.K., Jordan R., Lo M.K., Ray A.S., Mackman R.L., Soloveva V., Siegel D., Perron M., Bannister R., Hui H.C. (2016). Therapeutic Efficacy of the Small Molecule GS-5734 against Ebola Virus in Rhesus Monkeys. Nature.

[66] Mulangu S., Dodd L.E., Davey R.T., Tshiani Mbaya O., Proschan M., Mukadi D., Lusakibanza Manzo M., Nzolo D., Tshomba Oloma A., Ibanda A. (2019). A Randomized, Controlled Trial of Ebola Virus Disease Therapeutics. N. Engl. J. Med..

[67] Yin W., Mao C., Luan X., Shen D.-D., Shen Q., Su H., Wang X., Zhou F., Zhao W., Gao M. (2020). Structural Basis for Inhibition of the RNA-Dependent RNA Polymerase from SARS-CoV-2 by Remdesivir. Science.

[68] Malin J.J., Suárez I., Priesner V., Fätkenheuer G., Rybniker J. (2020). Remdesivir against COVID-19 and Other Viral Diseases. Clin. Microbiol. Rev..

[69] Brown A.J., Won J.J., Graham R.L., Dinnon K.H., Sims A.C., Feng J.Y., Cihlar T., Denison M.R., Baric R.S., Sheahan T.P. (2019). Broad Spectrum Antiviral Remdesivir Inhibits Human Endemic and Zoonotic Deltacoronaviruses with a Highly Divergent RNA Dependent RNA Polymerase. Antivir. Res..

[70] Frediansyah A., Nainu F., Dhama K., Mudatsir M., Harapan H. (2021). Remdesivir and Its Antiviral Activity against COVID-19: A Systematic Review. Clin. Epidemiol. Glob. Health.

[71] Pizzorno A., Padey B., Dubois J., Julien T., Traversier A., Dulière V., Brun P., Lina B., Rosa-Calatrava M., Terrier O. (2020). In Vitro Evaluation of Antiviral Activity of Single and Combined Repurposable Drugs against SARS-CoV-2. Antivir. Res..

[72] Spinner C.D., Gottlieb R.L., Criner G.J., Arribas López J.R., Cattelan A.M., Soriano Viladomiu A., Ogbuagu O., Malhotra P., Mullane K.M., Castagna A. (2020). Effect of Remdesivir vs Standard Care on Clinical Status at 11 Days in Patients With Moderate COVID-19: A Randomized Clinical Trial. JAMA.

[73] Zhou P., Yang X.-L., Wang X.-G., Hu B., Zhang L., Zhang W., Si H.-R., Zhu Y., Li B., Huang C.-L. (2020). A Pneumonia Outbreak Associated with a New Coronavirus of Probable Bat Origin. Nature.

[74] Wang Y., Zhang D., Du G., Du R., Zhao J., Jin Y., Fu S., Gao L., Cheng Z., Lu Q. (2020). Remdesivir in Adults with Severe COVID-19: A Randomised, Double-Blind, Placebo-Controlled, Multicentre Trial. Lancet.

[75] De Vito A., Poliseno M., Colpani A., Zauli B., Puci M.V., Santantonio T., Meloni M.C., Fois M., Fanelli C., Saderi L. (2022). Reduced Risk of Death in People with SARS-CoV-2 Infection Treated with Remdesivir: A Nested Case–Control Study. Curr. Med. Res. Opin..

[76] Veklury® (Remdesivir) Retains Antiviral Activity Against Omicron, Delta and Other Emergent SARS-CoV-2 Variants in Multiple In Vitro Studies. https://www.gilead.com/news-and-press/press-room/press-releases/2022/2/veklury-remdesivir-retains-antiviral-activity-against-omicron-delta-and-other-emergent-sarscov2-variants-in-multiple-in-vitro-studies.

[77] Ramos-Rincon J.-M., López-Carmona M.-D., Cobos-Palacios L., López-Sampalo A., Rubio-Rivas M., Martín-Escalante M.-D., de-Cossio-Tejido S., Taboada-Martínez M.-L., Muiño-Miguez A., Areses-Manrique M. (2022). Remdesivir in Very Old Patients (≥80 Years) Hospitalized with COVID-19: Real World Data from the SEMI-COVID-19 Registry. J. Clin. Med..

[78] Gao Y., Ding M., Dong X., Zhang J., Kursat Azkur A., Azkur D., Gan H., Sun Y., Fu W., Li W. (2021). Risk Factors for Severe and Critically Ill COVID-19 Patients: A Review. Allergy.

[79] Zhou F., Yu T., Du R., Fan G., Liu Y., Liu Z., Xiang J., Wang Y., Song B., Gu X. (2020). Clinical Course and Risk Factors for Mortality of Adult Inpatients with COVID-19 in Wuhan, China: A Retrospective Cohort Study. Lancet.

[80] Jayk Bernal A., Gomes da Silva M.M., Musungaie D.B., Kovalchuk E., Gonzalez A., Delos Reyes V., Martín-Quirós A., Caraco Y., Williams-Diaz A., Brown M.L. (2022). Molnupiravir for Oral Treatment of COVID-19 in Nonhospitalized Patients. N. Engl. J. Med..

[81] Mohd I.., Kumar Arora M., Asdaq S.M.B., Khan S.A., Alaqel S.I., Alshammari M.K., Alshehri M.M., Alshrari A.S., Mateq Ali A., Al-shammeri A.M. (2021). Discovery, Development, and Patent Trends on Molnupiravir: A Prospective Oral Treatment for COVID-19. Molecules.

[82] Tian L., Pang Z., Li M., Lou F., An X., Zhu S., Song L., Tong Y., Fan H., Fan J. (2022). Molnupiravir and Its Antiviral Activity Against COVID-19. Front. Immunol..

[83] Gordon C.J., Tchesnokov E.P., Schinazi R.F., Götte M. (2021). Molnupiravir Promotes SARS-CoV-2 Mutagenesis via the RNA Template. J. Biol. Chem..

[84] Kabinger F., Stiller C., Schmitzová J., Dienemann C., Kokic G., Hillen H.S., Höbartner C., Cramer P. (2021). Mechanism of Molnupiravir-Induced SARS-CoV-2 Mutagenesis. Nat. Struct. Mol. Biol..

[85] Maisonnasse P., Aldon Y., Marc A., Marlin R., Dereuddre-Bosquet N., Kuzmina N.A., Freyn A.W., Snitselaar J.L., Gonçalves A., Caniels T.G. (2021). COVA1-18 Neutralizing Antibody Protects against SARS-CoV-2 in Three Preclinical Models. Nat. Commun..

[86] Sheahan T.P., Sims A.C., Zhou S., Graham R.L., Pruijssers A.J., Agostini M.L., Leist S.R., Schäfer A., Dinnon K.H., Stevens L.J. (2020). An Orally Bioavailable Broad-Spectrum Antiviral Inhibits SARS-CoV-2 in Human Airway Epithelial Cell Cultures and Multiple Coronaviruses in Mice. Sci. Transl. Med..

[87] Troth S., Butterton J., DeAnda C.S., Escobar P., Grobler J., Hazuda D., Painter G. (2021). Letter to the Editor in Response to Zhou et Al. J. Infect. Dis..

[88] Abdelnabi R., Foo C.S., De Jonghe S., Maes P., Weynand B., Neyts J. (2021). Molnupiravir Inhibits Replication of the Emerging SARS-CoV-2 Variants of Concern in a Hamster Infection Model. J. Infect. Dis..

[89] Takashita E., Kinoshita N., Yamayoshi S., Sakai-Tagawa Y., Fujisaki S., Ito M., Iwatsuki-Horimoto K., Chiba S., Halfmann P., Nagai H. (2022). Efficacy of Antibodies and Antiviral Drugs against COVID-19 Omicron Variant. N. Engl. J. Med..

[90] Rosenke K., Okumura A., Lewis M.C., Feldmann F., Meade-White K., Bohler W.F., Griffin A., Rosenke R., Shaia C., Jarvis M.A. (2022). Molnupiravir Inhibits SARS-CoV-2 Variants Including Omicron in the Hamster Model. JCI Insight.

[91] De Vito A., Colpani A., Bitti A., Zauli B., Meloni M.C., Fois M., Denti L., Bacciu S., Marcia C., Maida I. (2022). Safety and Efficacy of Molnupiravir in SARS-CoV-2-infected Patients: A Real-life Experience. J. Med. Virol..

[92] Flisiak R., Zarębska-Michaluk D., Rogalska M., Kryńska J.A., Kowalska J., Dutkiewicz E., Dobrowolska K., Jaroszewicz J., Moniuszko-Malinowska A., Rorat M. (2022). Real-World Experience with Molnupiravir during the Period of SARS-CoV-2 Omicron Variant Dominance. Pharmacol. Rep..

[93] Gonda K., Suzuki K., Kono K., Takenoshita S. (2022). Safety of Oral Administration of Molnupiravir for Hospitalized Elderly People Aged 80 Years Old or Older with COVID-19. Res. Sq..

[94] Joshi S., Parkar J., Ansari A., Vora A., Talwar D., Tiwaskar M., Patil S., Barkate H. (2021). Role of Favipiravir in the Treatment of COVID-19. Int. J. Infect. Dis..

[95] Basu D., Chavda V.P., Mehta A.A. (2022). Therapeutics for COVID-19 and Post COVID-19 Complications: An Update. Curr. Res. Pharmacol. Drug Discov..

[96] Hassanipour S., Arab-Zozani M., Amani B., Heidarzad F., Fathalipour M., Martinez-de-Hoyo R. (2021). The Efficacy and Safety of Favipiravir in Treatment of COVID-19: A Systematic Review and Meta-Analysis of Clinical Trials. Sci Rep.

[97] Cai Q., Yang M., Liu D., Chen J., Shu D., Xia J., Liao X., Gu Y., Cai Q., Yang Y. (2020). Experimental Treatment with Favipiravir for COVID-19: An Open-Label Control Study. Engineering.

[98] McMahon J.H., Lau J.S.Y., Coldham A., Roney J., Hagenauer M., Price S., Bryant M., Garlick J., Paterson A., Lee S.J. (2022). Favipiravir in Early Symptomatic COVID-19, a Randomised Placebo-Controlled Trial. eClinicalMedicine.

[99] Udwadia Z.F., Singh P., Barkate H., Patil S., Rangwala S., Pendse A., Kadam J., Wu W., Caracta C.F., Tandon M. (2021). Efficacy and Safety of Favipiravir, an Oral RNA-Dependent RNA Polymerase Inhibitor, in Mild-to-Moderate COVID-19: A Randomized, Comparative, Open-Label, Multicenter, Phase 3 Clinical Trial. Int. J. Infect. Dis..

[100] Yuan Y., Lu Q.-B., Yao W.-S., Zhao J., Zhang X.-A., Cui N., Yuan C., Yang T., Peng X.-F., Lv S.-M. (2021). Clinical Efficacy and Safety Evaluation of Favipiravir in Treating Patients with Severe Fever with Thrombocytopenia Syndrome. eBioMedicine.

[101] Papp H., Lanszki Z., Keserű G.M., Jakab F. (2022). Favipiravir for the Treatment of COVID-19 in Elderly Patients—What Do We Know after 2 Years of COVID-19?. GeroScience.

[102] Ivashchenko A.A., Dmitriev K.A., Vostokova N.V., Azarova V.N., Blinow A.A., Egorova A.N., Gordeev I.G., Ilin A.P., Karapetian R.N., Kravchenko D.V. (2021). AVIFAVIR for Treatment of Patients With Moderate Coronavirus Disease 2019 (COVID-19): Interim Results of a Phase II/III Multicenter Randomized Clinical Trial. Clin. Infect. Dis..

[103] Shinkai M., Tsushima K., Tanaka S., Hagiwara E., Tarumoto N., Kawada I., Hirai Y., Fujiwara S., Komase Y., Saraya T. (2021). Efficacy and Safety of Favipiravir in Moderate COVID-19 Pneumonia Patients without Oxygen Therapy: A Randomized, Phase III Clinical Trial. Infect. Dis. Ther..

[104] Karatas E., Aksoy L., Ozaslan E. (2021). Association of Early Favipiravir Use with Reduced COVID-19 Fatality among Hospitalized Patients. Infect. Chemother..

[105] Wang Y., Fan G., Salam A., Horby P., Hayden F.G., Chen C., Pan J., Zheng J., Lu B., Guo L. (2020). Comparative Effectiveness of Combined Favipiravir and Oseltamivir Therapy Versus Oseltamivir Monotherapy in Critically Ill Patients With Influenza Virus Infection. J. Infect. Dis..

[106] Bureau E.N. Russian Drug Avifavir Effective against Corona Virus Variants: ChemRar Group. Express Pharma.

[107] Pilkington V., Pepperrell T., Hill A. (2020). A Review of the Safety of Favipiravir—A Potential Treatment in the COVID-19 Pandemic?. J. Virus Erad..

[108] Raez L.E., Papadopoulos K., Ricart A.D., Chiorean E.G., Dipaola R.S., Stein M.N., Rocha Lima C.M., Schlesselman J.J., Tolba K., Langmuir V.K. (2013). A Phase I Dose-Escalation Trial of 2-Deoxy-D-Glucose Alone or Combined with Docetaxel in Patients with Advanced Solid Tumors. Cancer Chemother. Pharmacol..

[109] Samal K.C., Panda B., Behera L. (2021). Anti-Covid Drug: 2-Deoxy-D-Glucose and Its Mechanism of Action. Biot. Res. Today.

[110] Kalyanaraman B. (2021). Reactive Oxygen Species, Proinflammatory and Immunosuppressive Mediators Induced in COVID-19: Overlapping Biology with Cancer. RSC Chem. Biol..

[111] Bhatt A.N., Kumar A., Rai Y., Kumari N., Vedagiri D., Harshan K.H., Chinnadurai V., Chandna S. (2022). Glycolytic Inhibitor 2-Deoxy-d-Glucose Attenuates SARS-CoV-2 Multiplication in Host Cells and Weakens the Infective Potential of Progeny Virions. Life Sci..

[112] Ogando N.S., Dalebout T.J., Zevenhoven-Dobbe J.C., Limpens R.W.A.L., van der Meer Y., Caly L., Druce J., de Vries J.J.C., Kikkert M., Bárcena M. (2020). SARS-Coronavirus-2 Replication in Vero E6 Cells: Replication Kinetics, Rapid Adaptation and Cytopathology. J. Gen. Virol..

[113] Balkrishna A., Thakur P., Singh S., Dev S., Jain V., Varshney A., Sharma R. (2021). Glucose Antimetabolite 2-Deoxy-D-Glucose and Its Derivative as Promising Candidates for Tackling COVID-19: Insights Derived from in Silico Docking and Molecular Simulations. Authorea.

[114] Samui P., Mondal J., Ahmad B., Chatterjee A.N. (2022). Clinical Effects of 2-DG Drug Restraining SARS-CoV-2 Infection: A Fractional Order Optimal Control Study. J. Biol. Phys..

[115] Verma A., Adhikary A., Woloschak G., Dwarakanath B.S., Papineni R.V.L. (2020). A Combinatorial Approach of a Polypharmacological Adjuvant 2-Deoxy-D-Glucose with Low Dose Radiation Therapy to Quell the Cytokine Storm in COVID-19 Management. Int. J. Radiat. Biol..

[116] www.ETHealthworld.com. Indian Drug 2DG Can Reduce Heart Damage by Coronavirus, Find US Researchers—ET HealthWorld. https://health.economictimes.indiatimes.com/news/pharma/indian-drug-2dg-can-reduce-heart-damage-by-coronavirus-find-us-researchers/95382862.

[117] DCGI Approves Anti-COVID Drug Developed by DRDO for Emergency Use. https://pib.gov.in/pib.gov.in/Pressreleaseshare.aspx?PRID=1717007.

[118] Bhatt A.N., Shenoy S., Munjal S., Chinnadurai V., Agarwal A., Vinoth Kumar A., Shanavas A., Kanwar R., Chandna S. (2022). 2-Deoxy-d-Glucose as an Adjunct to Standard of Care in the Medical Management of COVID-19: A Proof-of-Concept and Dose-Ranging Randomised Phase II Clinical Trial. BMC Infect. Dis..

[119] Panagopoulos P., Petrakis V., Trypsianis G., Papazoglou D. (2022). Early 3-Day Course of Remdesivir in Vaccinated Outpatients with SARS-CoV-2 Infection. A Success Story. J. Chemother..

[120] Khoo S.H., FitzGerald R., Saunders G., Middleton C., Ahmad S., Edwards C.J., Hadjiyiannakis D., Walker L., Lyon R., Shaw V. (2023). Molnupiravir versus Placebo in Unvaccinated and Vaccinated Patients with Early SARS-CoV-2 Infection in the UK (AGILE CST-2): A Randomised, Placebo-Controlled, Double-Blind, Phase 2 Trial. Lancet Infect. Dis..

[121] Herman B., Wong M.C., Viwattanakulvanid P. (2022). Vaccination Status, Favipiravir, and Micronutrient Supplementation Roles in Post-COVID Symptoms: A Longitudinal Study. PLoS ONE.

